# The differential short- and long-term effects of HIV-1 latency-reversing agents on T cell function

**DOI:** 10.1038/srep30749

**Published:** 2016-08-02

**Authors:** G. Clutton, Y. Xu, P. L. Baldoni, K. R. Mollan, J. Kirchherr, W. Newhard, Kara Cox, J. D. Kuruc, A. Kashuba, R. Barnard, N. Archin, C. L. Gay, M. G. Hudgens, D. M. Margolis, N. Goonetilleke

**Affiliations:** 1 Department of Microbiology & Immunology, UNC Chapel Hill School of Medicine, Chapel Hill, North Carolina, USA; 2 Department of Biostatistics, UNC Chapel Hill, Chapel Hill, North Carolina, USA; 3 Lineberger Comprehensive Care Center, UNC Chapel Hill, Chapel Hill, North Carolina, USA; 4 Department of Medicine and UNC HIV Cure Center, UNC Chapel Hill School of Medicine, Chapel Hill, North Carolina, USA; 5 Merck Research Laboratories, White Horse Junction, Pennsylvania, USA; 6 Eshelman School of Pharmacy, UNC Chapel Hill, North Carolina, USA

## Abstract

Despite the extraordinary success of HIV-1 antiretroviral therapy in prolonging life, infected individuals face lifelong therapy because of a reservoir of latently-infected cells that harbor replication competent virus. Recently, compounds have been identified that can reverse HIV-1 latency *in vivo*. These latency- reversing agents (LRAs) could make latently-infected cells vulnerable to clearance by immune cells, including cytolytic CD8+ T cells. We investigated the effects of two leading LRA classes on CD8+ T cell phenotype and function: the histone deacetylase inhibitors (HDACis) and protein kinase C modulators (PKCms). We observed that relative to HDACis, the PKCms induced much stronger T cell activation coupled with non-specific cytokine production and T cell proliferation. When examining antigen-specific CD8+ T cell function, all the LRAs except the HDACi Vorinostat reduced, but did not abolish, one or more measurements of CD8+ T cell function. Importantly, the extent and timing of these effects differed between LRAs. Panobinostat had detrimental effects within 10 hours of drug treatment, whereas the effects of the other LRAs were observed between 48 hours and 5 days. These observations suggest that scheduling of LRA and CD8+ T cell immunotherapy regimens may be critical for optimal clearance of the HIV-1 reservoir.

A major advance in HIV-1 eradication strategies has been the identification of compounds in different drug classes that can reactivate HIV-1 in latently-infected cells. These putative latency-reversing agents (LRAs) include histone deacetylase inhibitors (HDACis) that can induce viral transcription from the HIV-1 LTR promoter by disrupting the repressive chromatin state induced by histone deacetylases^1^,^2^, and protein kinase C modulators (PKCms) that can trigger viral RNA expression by activating transcription factors downstream of protein kinase C^3^,^4^,^5^. Promisingly, three HDACis, Vorinostat, Romidepsin, and Panobinostat, have increased plasma viral RNA in HIV-1-infected, durably suppressed individuals, suggesting successful HIV-1 reactivation^6^,^7^,^8^,^9^. However, consistent with *in vitro* studies demonstrating that reactivated cells do not die from viral cytopathic effects^10^, HDACi dosing of participants did not produce decreases in cell-associated viral DNA. These observations suggest that, alone, LRAs do not reduce the size of the HIV-1 reservoir and that additional strategies will be needed to purge latently-infected cells that have been reactivated.

Recent studies have demonstrated that HIV-1-specific CD8+ T cells can detect and eliminate latently-infected cells following *in vitro* latency reversal^10^,^11^,^12^. Deng and colleagues reported that despite detectable T cell escape mutations in proviral DNA, combination anti-retroviral (cART)-treated chronically HIV-1-infected individuals retained CD8+ T cell clones capable of eliminating autologous infected CD4+ T cells^12^. The authors also showed that the clearance of infected CD4+ T cells was further enhanced by prior expansion of CD8+ T cells targeting conserved HIV-1 epitopes. These observations provide a clear rationale for using latency-reversing agents in combination with immunotherapeutic strategies to boost the endogenous HIV-1-specific CD8+ T cell response and/or redirect the response toward conserved epitopes. However, since both HDACis and PKCms have been reported to have immunomodulatory effects^13^,^14^,^15^,^16^, it is important to consider whether these agents may impact the ability of CD8+ T cells to respond to viral antigen. Recently, Jones *et al.* reported that HDACis impaired HIV-1-specific CD8+ T cell responses *in vitro*^17^, but this was not observed by Sung *et al.*^11^, and in clinical studies there was no reduction in the frequency of cytokine-producing HIV-1-specific CD8+ T cells following multiple doses of Vorinostat or Romidepsin^7^,^8^. PKCms have been reported to induce HIV-1 reactivation without proliferation or cytokine production in resting CD4+ T cells^18^,^19^, but given that PKC activity is central to T cell receptor-mediated signaling^20^, further studies are needed to elucidate their impacts on both antigen presentation and effector functions of T cells.

In this study, we undertook a comprehensive analysis of the effects of clinically relevant doses and exposure periods to HDACis and PKCms on T cell activation, cytokine production, production of cytotoxic effectors, and proliferation, using PBMC from HIV-1-infected participants durably suppressed on cART. We also measured T cell activation and function *ex vivo* from participants receiving oral Vorinostat. We report that changes in T cell phenotype and function were significantly greater and more sustained in PBMC treated with PKCms compared to HDACis, but that even within the same class, compounds differed in their effects. Interestingly, some effects were only evident 48 hours or more after a short 3 hour exposure to drug. We conclude that the timing of antigen presentation by reactivated cells will be critical in determining whether their clearance by CD8+ T cells is impaired following treatment with a LRA.

## Results

### HDACis minimally activate T cells

**Figure 1:**
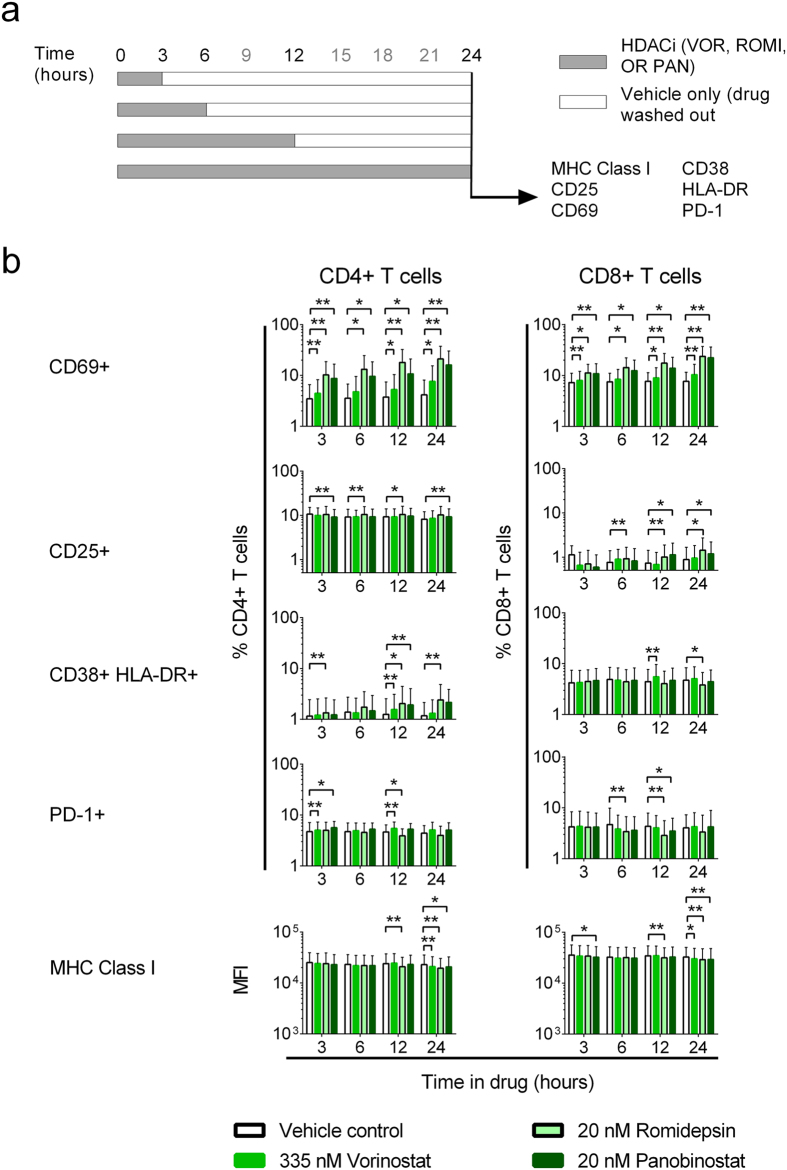
HDACis differentially activate T cells. (**a**) Experimental design. PBMC from HIV-1-seropositive participants durably suppressed on cART (n = 7) were exposed to vehicle (0.5% DMSO) or LRA for 3, 6, 12, or 24 hours, washed to remove extracellular drug, and maintained in vehicle for the remaining culture period. Activation marker expression was assessed at 24 hours. (**b**) Expression of activation markers on CD4+ and CD8+ T cells at 24 hours. Measurements were compared between vehicle and HDACi treatment using an exact Wilcoxon Signed Rank test. *p < 0.05; **p ≤ 0.016.

The plasma half-lives of Vorinostat, Romidepsin, and Panobinostat *in vivo* are reported as approximately 2 hours, 3.5 hours, and 30 hours, respectively^21^,^22^,^23^. To mimic *in vivo* exposure of cells to drug we tested the effect of different periods of exposure, 3, 6, 12, or 24 hours, to Vorinostat, Romidepsin, and Panobinostat on *ex vivo* T cell activation. The concentration used for each drug was determined by previously reported *in vivo* plasma C_max_ levels that also elicited HIV-1 reactivation *in vitro*^6^,^9^,^24^,^25^. PBMC were also exposed to PHA/IL-2, which induces generalized T cell activation through cross-linking of T cell receptors and cytokine signaling. After exposure, cells were washed to remove free drug and were maintained in 0.5% DMSO (vehicle) for the remaining culture period (Fig. 1a). All measurements were made at 24 hours. LC/MS-MS methods confirmed that washing effectively removed drugs from the cell cultures (Table 1).

In the cell cultures exposed to vehicle only, levels of activation markers remained stable between 3 and 24 hours (Fig. 1b, Supplementary Fig. S1). The effects on cellular activation following exposure of PBMC to the HDACis were modest when compared with PHA/IL-2, which induced significant increases in the frequencies of all activation markers as well as surface levels of MHC Class I (Supplementary Fig. S2). HDACi exposure had the greatest effect on the highly sensitive, early activation marker CD69 (Fig. 1b), with significant increases observed in both CD4+ and CD8+ T cells after a 3 hour exposure. Romidepsin had the largest effect (3-fold increase on CD4+ T cells and 1.6-fold on CD8+ T cells; p < 0.05 by exact Wilcoxon Signed Rank test), followed by Panobinostat, then Vorinostat (1.3-fold mean increase on CD4+ T cells and 1.1-fold on CD8+ T cells; p < 0.05). HDACi exposure also induced small but consistent increases in the frequency of CD25 and CD38/HLA-DR-expressing CD8+ T cells, the frequency of PD1 positive CD4+ T cells, and a decrease in MHC Class I mean fluorescence intensity (MFI). These effects were typically only observed after 6 or more hours of exposure of cells to drug (Fig. 1b).

HDACis did not substantially alter the frequency of memory (CD45RO+) CD4+ or CD8+ T cells; similarly the activation of memory T cells mirrored those observed for the bulk T cell populations (Supplementary Fig. S3).

### The PKC modulator Ingenol 3,20-dibenzoate strongly activates T cells

To investigate whether PKCms differed from HDACis in their ability to activate T cells, we repeated the drug washout experiment using Ingenol 3,20-dibenzoate (Ingenol-db), which can activate HIV-1 expression in resting primary CD4+ T cells from HIV-1-infected individuals on cART^26^. Measuring PKCm pharmacokinetics is challenging^27^ and there are limited data on the half-life of this drug *in vivo*. We therefore used the same exposure periods as described above to enable a direct comparison with the HDACis, again using PBMC from HIV-1-infected participants durably suppressed on cART. A 3 hour exposure to Ingenol-db induced T cell activation to levels comparable to or greater than that of PHA/IL-2, with over 90% of CD4+ T cells and 85% of CD8+ T cells expressing CD69 at 24 hours (p < 0.05 by exact Wilcoxon Signed Rank test; Supplementary Fig. S2). The frequency of CD25-expressing cells and MHC Class I levels were significantly increased on both CD4+ and CD8+ T cell subsets (p < 0.05) (Supplementary Fig. S2).

### PKCms induce sustained increases in T cell activation

**Figure 2:**
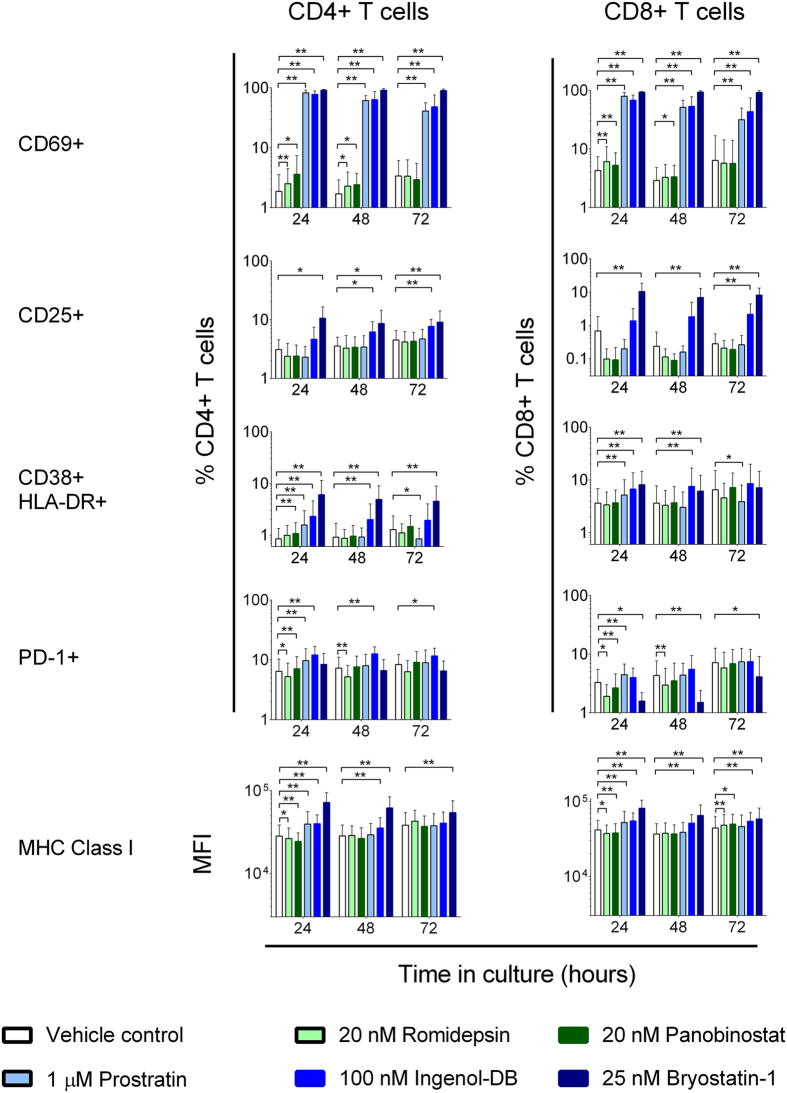
PKCms potently activate T cells. Expression of activation markers at 24, 48, and 72 hours on CD4+ and CD8+ T cells. PBMC from HIV-1-seropositive participants durably suppressed on cART (n = 8) were exposed to vehicle (0.5% DMSO), HDACis or PKCms for 3 hours, washed to remove extracellular drug, and maintained in vehicle for the remainder of the 72 hour culture period. Measurements were compared between vehicle and LRA treatment using an exact Wilcoxon Signed Rank test. *p < 0.05; **p ≤ 0.016.

Having observed that HDACis and the PKCm Ingenol-db induced differing levels of T cell activation at 24 hours, we next assessed the longevity of these effects. PBMC from eight HIV-1-seropositive participants durably suppressed on cART were exposed to LRA for 3 or 6 hours, washed as before, and T cell activation measured at 24, 48, and 72 hours. Due to its modest effects in the original experiments, Vorinostat was omitted and two further PKCms, Prostratin and Bryostatin-1, were included. Like Ingenol-db, both of these PKCms can reactivate HIV-1 in primary resting CD4+ T cells^18^,^28^. A 3 hour exposure to the PKCms increased CD69 levels on the majority of CD4+ and CD8+ T cells, which was maintained over 72 hours (Fig. 2). Similar effects were observed on CD4+ and CD8+ T cells from HIV-1-seronegative participants (Supplementary Fig. S4). The PKCms also induced significant increases in CD25, co-expression of CD38 and HLA-DR, PD-1, and MHC Class I that were sustained over 48 to 72 hours. Overall, Bryostatin-1 induced the largest and most sustained increases in activation marker expression, followed by Ingenol-db and then Prostratin. These effects was comparable to those observed for PHA/IL-2 (Supplementary Fig. S5). In contrast, the relatively modest increases in CD69 expression induced by Romidepsin and Panobinostat at 24 hours were no longer detected at 72 hours.

Bryostatin-1 reduced the expression of the memory marker CD45RO on CD4+ and CD8+ T cells at 24 hours, but these differences were not sustained over 72 hours, and memory (CD45RO+) T cells exhibited similar phenotypic changes to bulk T cells (Supplementary Fig. S6). Exposure of PBMC to LRA for 6 hours produced broadly similar results to the 3 hour exposure (Supplementary Fig. S7).

In summary, the HDACis Romidepsin and Panobinostat induced minor and short-lived effects on T cell activation whereas PKCms induced clear and sustained cellular activation.

### LRAs have variable toxicity profiles

**Figure 3:**
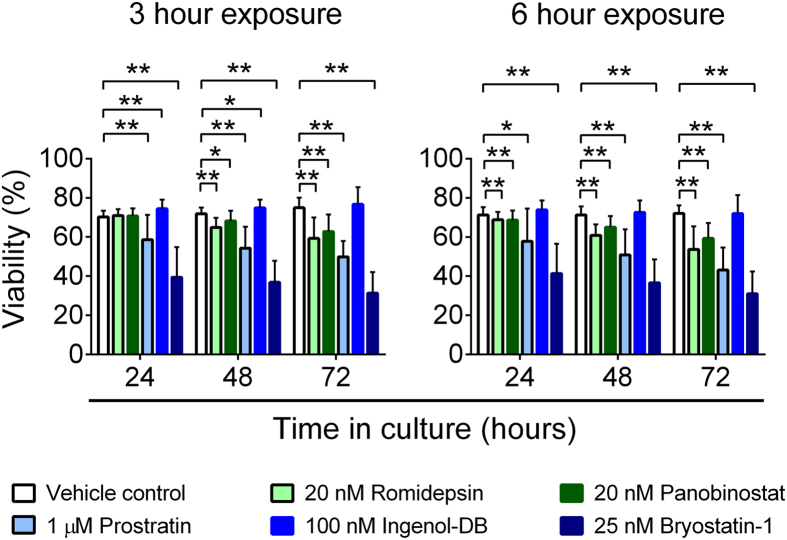
Toxicity of LRAs *in vitro*. Viability of PBMC from HIV-1-seropositive donors durably suppressed on cART (n = 8). PBMC were exposed to vehicle (0.5% DMSO) or LRAs for 3 (left) or 6 (right) hours, washed to remove extracellular drug, and maintained in vehicle for the remainder of the culture period. Viability was measured at 24, 48, and 72 hours. Measurements were compared between vehicle and HDACi treatment using an exact Wilcoxon Signed Rank test. *p < 0.05; **p ≤ 0.016.

Within the same experiments, an amine-reactive dye was used to assess the viability of PBMC from HIV-1-infected participants on cART. A 3 hour exposure to Romidepsin or Panobinostat did not affect viability compared to exposure to vehicle at 24 hours, but viability at 48 and 72 hours was significantly reduced (p < 0.05 by exact Wilcoxon Signed Rank test) (Fig. 3). After a longer exposure period of 6 hours, Romidepsin and Panobinostat treatment reduced PBMC viability at all time points (p < 0.01). When we examined the effects of the PKCms, PBMC exposed to Ingenol-db for 3 but not 6 hours exhibited modestly but significantly increased PBMC viability at 24 and 48 hours compared to vehicle-treated PBMC (p < 0.05), suggesting some effects on cell division (see below). In contrast, exposure to Prostratin or Bryostatin-1 (either 3 or 6 hr) significantly reduced viability at all subsequent time points (p < 0.01) (Fig. 3). Therefore, the effects of different LRAs on viability and activation depended on both the period of exposure to drug and the duration of the culture period, with Bryostatin-1 the most toxic, followed by Prostratin, Romidepsin, and Panobinostat.

### LRAs have differential effects on pro-inflammatory cytokine release

**Figure 4:**
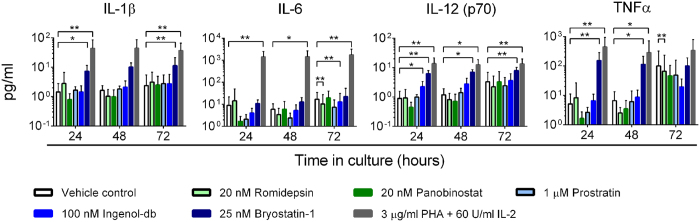
Cytokine release following PBMC exposure to LRAs. Cytokine concentrations in culture supernatants of PBMC from HIV-1-seropositive participants durably suppressed on cART (n = 5–8). PBMC were exposed to vehicle (0.5% DMSO) or LRAs for 3 hours, washed to remove extracellular drug, and maintained in vehicle for the remainder of the culture period. Supernatants were collected at 24, 48, and 72 hours. *p < 0.05; **p ≤ 0.016.

We next investigated whether LRAs promoted pro-inflammatory cytokine release. Three hours exposure to Vorinostat, Panobinostat, or Romidepsin did not significantly alter cytokine levels in supernatants collected at 24 hours relative to exposure to vehicle (Supplementary Fig. S8). Similarly, no increases in cytokine levels were observed in culture supernatants collected 48 and 72 hours after either Romidepsin or Panobinostat exposure (Fig. 4 and Supplementary Fig. S9).

In contrast to the HDACis, Bryostatin-1 significantly increased the concentration of IL-1β, IL-12 (p70), and TNFα, but not IL-6, relative to treatment with vehicle. These increases were however modest compared to those observed after exposure to PHA/IL-2 (Fig. 4). For example, for IL-1β a mean 5-fold increase relative to vehicle was observed in Bryostatin-exposed supernatants compared to a 30-fold increase in PHA/IL-2-exposed supernatants at 24 hours (Fig. 4). Bryostatin-1, Ingenol-db, and Prostratin also modestly but significantly increased IFN-γ levels in supernatants at 24 hours (Supplementary Fig. S9). Similar results were observed for supernatants from cultures exposed to LRA for 6 hours (Supplementary Fig. S9).

### PKCms induce significant non-specific cytokine production by CD4+ and CD8+ T cells

**Figure 5:**
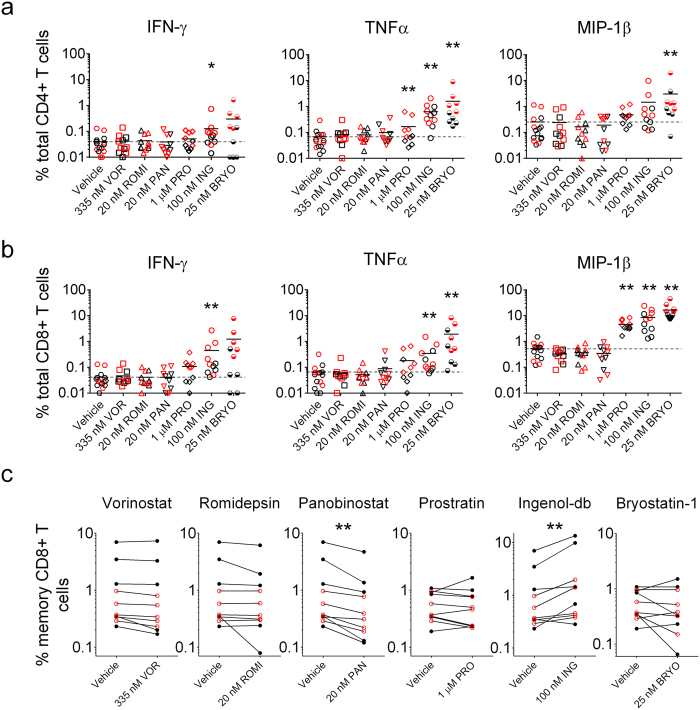
Effects of LRAs on non-specific and antigen-specific T cell function. PBMC from HIV-1-seropositive participants durably suppressed on cART (red symbols; n = 5) and HIV-1-seronegative participants (black symbols; n = 5) were exposed to vehicle (0.5% DMSO) or a LRA for 4 hours, washed and then cultured for a further 6 hours in either vehicle (to assess non-specific responses) or in the presence of peptides (to assess antigen-specific responses). (**a,b**) Non-specific cytokine production by CD4+ and CD8+ T cells. Dashed lines indicate mean responses in vehicle-exposed cultures. (**c**) The effect of 4 hours pre-exposure to LRAs on the frequency of memory CD8+ T cells degranulating (perforin^low^ CD107a+) and producing IFN-γ in response to peptide stimulation. PBMC from HIV-1-seropositive participants were exposed to HIV-1 CD8+ optimal epitopes, and PBMC from HIV-1-seronegative participants were exposed to a pool of ‘Flu, EBV, and CMV (FEC) CD8+T cell epitopes. Due to sample availability, not all LRAs were tested in all participants. Measurements were compared between vehicle and LRA treatment using an exact Stratified Wilcoxon Signed Rank test. *p < 0.05; **p ≤ 0.016.

We also assessed whether LRAs affected the production of cytokines and lytic molecules by T cells in either the presence or absence of peptide stimulation. T cell cytolysis is tightly controlled through MHC-TCR signaling^29^; however, agents such as LRAs may have “off-target” effects that dysregulate this process, impacting cellular function. PBMC from 10 participants (5 HIV-1-seropositive participants durably suppressed on cART and 5 seronegative participants) were exposed to Vorinostat, Romidepsin, Panobinostat, Prostratin, Ingenol-db, or Bryostatin-1 for 4 hours, washed to remove extracellular drug, and maintained in culture for a further 6 hours. Production of IFN-γ, TNFα, and MIP-1β was assessed by intracellular cytokine staining (Supplementary Fig. S10). HDACis did not induce non-specific cytokine production by CD4+ or CD8+ T cells (Fig. 5a,b). In contrast, all PKCms induced non-specific production of at least one cytokine by CD4+ and CD8+ T cells (Fig. 5a,b). Bryostatin-1 was the most potent inducer of TNFα (mean 27-fold increase compared to vehicle for CD4+ T cells and 50-fold for CD8+ T cells; p < 0.01 by exact Wilcoxon Signed Rank test stratified by HIV-1 serostatus) and MIP-1β (mean 57-fold for CD4+ T cells and 65-fold for CD8+ T cells, p < 0.05). Only Ingenol-db increased non-specific IFN-γ production by CD4+ and CD8+ T cells relative to the vehicle control (6-fold for CD4+ T cells and 15-fold for CD8+ T cells, p < 0.05). In summary, PKCms, but not HDACis, induced non-specific cytokine production by T cells.

### HDACis and PKCms have limited effects on short-term antigen-specific CD8+ T cell cytokine and lytic molecule production

In the same set of experiments, we examined whether HDACis and PKCms impacted *ex vivo* antigen-specific CD8+ T cell function. Following stimulation, CD8+ T cells rapidly release pre-formed perforin-containing granules, resulting in a reduction in intracellular perforin and the accumulation of CD107a contained in the granule membrane at the cell surface^30^. To assess cytotoxic T cell function and maximize assay stringency, we gated on cells that produced IFN-γ in response to peptide stimulation and were CD107a+ and perforin^low ^31 (Supplementary Fig. S10). We observed that relative to vehicle, pre-exposure to Panobinostat modestly but consistently reduced the frequency of antigen-specific CD8+ T cells exhibiting cytotoxic potential in both seropositive and seronegative individuals (mean 1.4-fold (range 1.1–1.7) decrease in seropositive; 2.0-fold (range 1.4–2.7) decrease in seronegative; p = 0.002 by exact Wilcoxon Signed Rank test stratified by HIV-1 serostatus (Fig. 5c)). Ingenol-db, on the other hand, increased the frequency of antigen-specific perforin^low^ CD107a+ IFN-γ+ CD8+ T cells (mean 1.6-fold (range 1.2–2.4) increase in seropositive; 1.9-fold (range 0.8–2.9) in seronegative; p = 0.006). None of the other drugs tested significantly altered the magnitude of this T cell response.

When antigen-specific CD8+ T cell responses involving other combinations of cytokines were assessed, Panobinostat exposure consistently decreased antigen-specific CD8+ T cell responses. While Vorinostat had no effect on production of lytic markers, exposure modestly reduced the frequency of antigen-specific CD8+ T cell responses involving the production of TNFα (p < 0.05; Table 2). Romidepsin did not significantly affect any of the functional parameters studied. Among the PKCms, Prostratin did not affect antigen-specific CD8+ T cell responses. Ingenol-db and Bryostatin-1, while both strongly inducing non-specific cytokine production (Fig. 5a,b), also increased the frequency of CD8+ T cells producing IFN-γ and/or TNFα in response to antigen after subtraction of the non-specific response (Table 2). In summary, Panobinostat was the only HDACi, when administered at a physiologically-achievable dose that significantly impaired antigen-specific lytic responses in primary CD8+ T cells. Among the PKCms, Ingenol-db and Bryostatin-1 enhanced some antigen-specific T cell responses.

### HDACis and PKCms impair antigen-specific CD8+ T cell proliferation

Another key element of T cell function is the capacity to proliferate in response to antigen. PBMC from 10 participants (5 HIV-1-seropositive participants receiving cART and 5 seronegative participants) were pulsed with the proliferation-tracking dye CFSE, exposed to LRA or vehicle for 4 hours, and washed to remove extracellular drug. Cells were then stimulated with either peptide (to assess antigen-specific proliferation) or vehicle (to assess non-specific proliferation) for 5 days.

**Figure 6:**
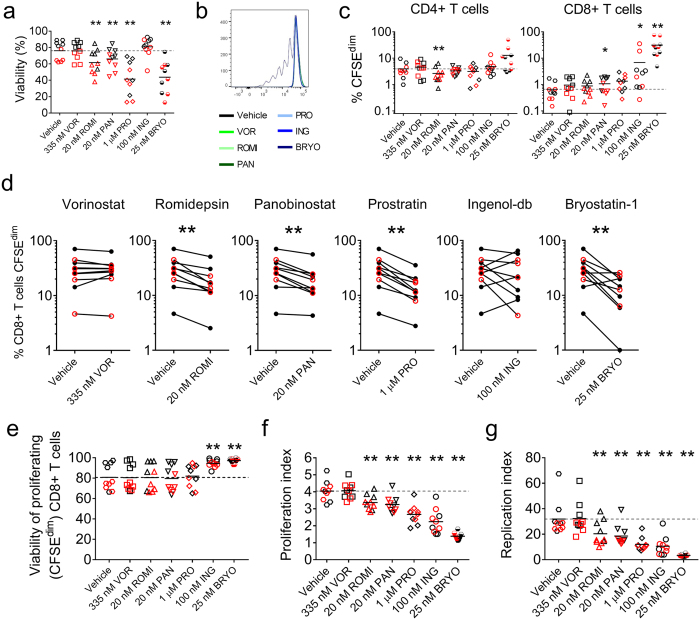
Effects of LRAs on T cell proliferation. PBMC from HIV-1-seropositive participants durably suppressed on cART (red symbols; n = 5) and HIV-1-seronegative participants (black symbols; n = 5) were exposed to vehicle (0.5% DMSO) or a LRA for 4 hours, washed and then cultured for 5 days in either vehicle (to assess non-specific proliferation) or in the presence of peptides (to assess antigen-specific proliferation). (**a**) PBMC viability measured at 5 days. (**b,c**) Representative histograms (**b**) and combined data (**c**) showing non-specific proliferation by CD4+ and CD8+ T cells exposed to vehicle or LRA for 4 hours. The frequency of proliferating (CFSE^dim^) cells was measured at 5 days. (**d**) The effect of 4 hours pre-exposure to LRA on (live) CD8+ T cell proliferation (% CFSE^dim^) in response to peptide, measured at 5 days. (**e**) Viability of all CD8+ T cells that had proliferated one or more times in response to peptide after 5 days. (**f**) The effect of 4 hours pre-exposure to LRA on the mean number of cycles of proliferation undergone by (live) antigen-stimulated cells over 5 days (proliferation index). (**g**) The effect of 4 hours pre-exposure to LRA on the mean number of (live) daughter cells generated by each antigen-specific parent cell over 5 days (replication index). Dashed lines indicate mean responses in vehicle-pre-exposed cultures. Measurements were compared between vehicle and LRA-treatment using an exact Stratified Wilcoxon Signed Rank test. *p < 0.05; **p ≤ 0.016.

We first examined whether LRA exposure impacted PBMC viability over the longer 5 day culture period. Vorinostat and Ingenol-db had no impact on viability relative to the vehicle control. Panobinostat and Romidepsin reduced viability by a mean of 10 and 14 percentage points respectively (p < 0.01 by exact Wilcoxon Signed Rank test stratified by HIV-1 serostatus). The effects of Prostratin and Bryostatin-1 were even greater and more variable; mean PBMC viability decreased by 35 percentage points for Prostratin and 32 percentage points for Bryostatin-1 (p < 0.01; Fig. 6a).

Vorinostat and Prostratin had no effect on non-specific T cell proliferation as measured by % CFSE^dim^ T cells (Fig. 6b,c). This contrasts with Bryostatin-1, which induced significant non-specific CD8+ T cell proliferation compared to the vehicle control, increasing the mean % CSFE^dim^ cells from 0.7% to 31% (p = 0.002); Fig. 6b,c). Ingenol-db also increased non-specific CD8+ T cell proliferation to a mean 7% (p = 0.023). A far more modest increase in non-specific CD8+ T cell proliferation was also observed following Panobinostat exposure. Interestingly, Romidepsin had no effect CD8+ T cells but reduced non-specific CD4+ T cell proliferation (Fig. 6c).

Within the same experiments, we assessed the effects LRA pre-exposure on antigen-specific proliferation, measuring the total frequency of CD8+ T cells that had proliferated one or more times in response to antigen (% CD8+ T cells CFSE^dim^), the mean number of divisions undertaken by proliferating (CFSE^dim^) cells (proliferation index), and the mean number of daughter cells generated by each antigen-specific precursor (replication index). Of the HDACis, Romidepsin and Panobinostat but not Vorinostat modestly reduced the total frequency of CD8+ T cells proliferating in response to antigen (p < 0.01 by exact Wilcoxon Signed Rank test stratified by HIV-1-serostatus) (Fig. 6d). The PKCms Prostratin and Bryostatin-1, but not Ingenol-db, also reduced the total frequency of proliferating antigen-specific CD8+ T cells (p < 0.05) (Fig. 6d). This was not the result of increased death of proliferating cells, as when we repeated the analysis gating first on total (both alive and dead) proliferating CD8+ T cells and then assessed their viability, we observed that none of the LRAs reduced proliferating cell viability, indeed Ingenol-db and Bryostatin-1-treated cultures harbored fewer dead cells that had proliferated (Fig. 6e). Rather, all LRAs except Vorinostat reduced the proliferation index of antigen-stimulated cells (Fig. 6f). Over 5 days, CD8+ T cells exposed to vehicle followed by antigen proliferated a mean of 4 times. Pre-treatment with Romidepsin or Panobinostat reduced the proliferation index to 3.4 and 3.3 respectively (p < 0.01). For PBMC pre-exposed to PKCms, Prostratin reduced the proliferation index to 2.8, Ingenol-db to 2.3, and Bryostatin-1 to 1.4 (p < 0.01). Romidepsin, Panobinostat, Prostratin, Ingenol-db, and Bryostatin-1 also reduced the replication index of antigen-stimulated CD8+ T cells (STATS; Fig. 6g). These observations suggested that these drugs induced cell cycle arrest of antigen-specific CD8+ T cells.

### *Ex vivo* effects of Vorinostat on T cell phenotype and function are minimal

**Figure 7:**
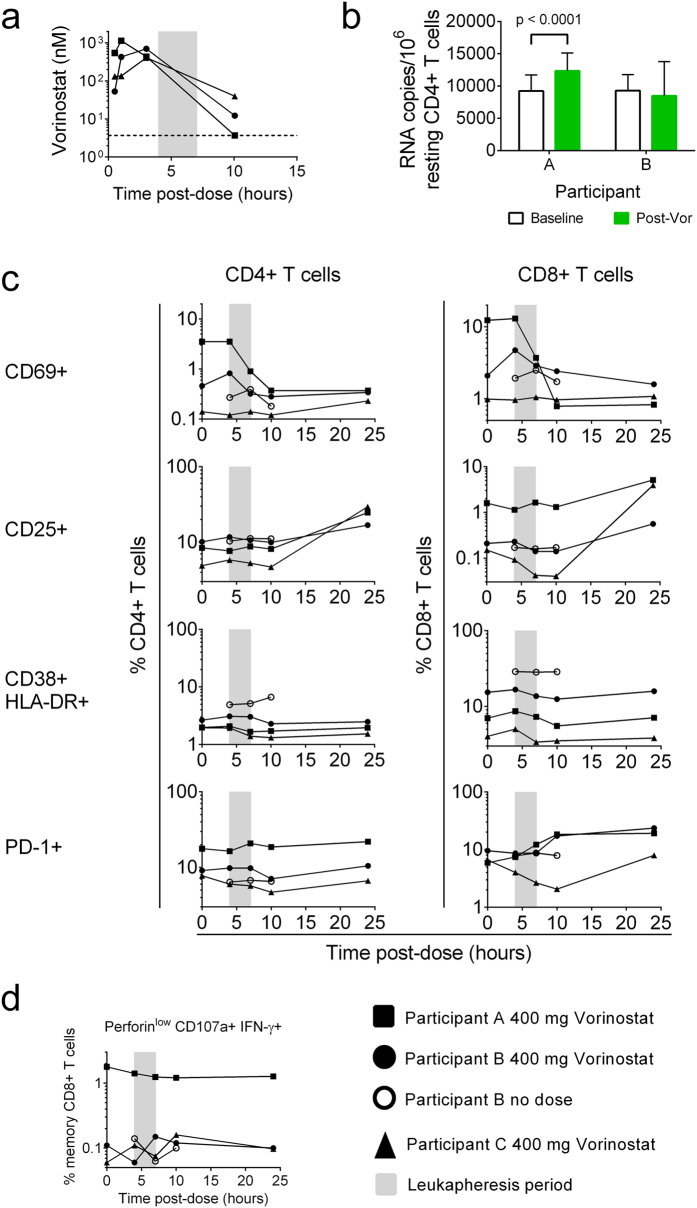
Effects of an oral dose of Vorinostat on T cell phenotype and function *ex vivo*. Participants received a single 400 mg oral dose of Vorinostat. (**a**) Vorinostat plasma pharmacokinetics in three participants 30 minutes to 10 hours after dosing. (**b**) Quantification of HIV-1 RNA in resting CD4+ T cells at baseline and following oral dosing with Vorinostat (data not available for participant C). (**c**) Expression of activation markers on peripheral blood CD4+ and CD8+ T cells immediately prior to dosing and 4, 7, 10 and 24 hours post-dose. (**d**) Frequency of memory CD8+ T cells in peripheral blood degranulating (perforin^low^ CD107a+) and producing IFN-γ in response to peptide stimulation pre-dose and 4, 7, 10, and 24 hours post-dose. Measurements of HIV-1 RNA in resting CD4+ T cells were compared between baseline and post-dose using a Mann-Whitney test.

We also examined the phenotype and function of T cells from three durably-suppressed HIV-1-seropositive donors who received a single 400 mg oral dose of Vorinostat. Plasma Vorinostat levels were monitored for 10 hours post-dose (Fig. 7a). PCR analysis using cells obtained by leukapheresis 4 hours after the dose indicated that Vorinostat had induced viral reactivation (defined as a significant increase in cell-associated viral RNA from baseline) in participant A but not participant B (no data available for participant C) (Fig. 7b). T cell activation was examined on cells freshly isolated from peripheral blood obtained immediately prior to the dose (time 0) and at 4, 7, 10, and 24 hours post-dose. Most markers were unchanged from baseline (Fig. 7c, Supplementary Fig. S11). However, we observed a consistent increase in the frequency of CD25-expressing CD4+ T cells (2.9-fold for participant A, 1.7-fold for B, and 6.0-fold for C) and CD8+ T cells (3.2-fold for participant A, 2.7-fold for B and 26.3-fold for C) 24 hours after the dose (compared to time 0). No other changes were consistent across the 3 subjects studied, though in 2 of 3 participants we observed increases in PD-1 frequencies, and in one participant there was a substantial decline in the frequency of circulating CD69+ T cells (9.5-fold for CD4+ T cells and 14.8-fold for CD8+ T cells) at 10 hours post-dose, maintained at 24 hours post-dose. These changes were not observed when this participant underwent leukapheresis without receiving Vorinostat on a previous visit (open circles, Fig. 7c), though on this visit we were unable to obtain a blood sample at 24 hours and so cannot exclude a possible effect of the leukapheresis procedure on the differences we observed 24 hours after Vorinostat dosing.

Memory (CD45RO+) T cells exhibited similar changes to those seen in the bulk T cell populations (Supplementary Fig. S12). We did not observe substantial changes in the frequency of circulating memory (CD45RO+) T cells (Supplementary Fig. S12), or the relative frequencies of naïve, central memory, effector memory, terminally differentiated, or resting CD4+ T cells (Supplementary Fig. S11).

CD8+ T cell function was also assessed, using cryopreserved PBMC, at the same time points. A modest (1.4-fold) decrease in the frequency of HIV-1-specific perforin^low^ CD107a+ IFN-γ+ CD8+ T cells was observed in participant A. In participants B and C, the T cell response was close to the cutoff for positivity (see Methods) at all time points and was of similar magnitude at 0 and 24 hours (Fig. 7d). While our numbers are low, these *ex vivo* data were consistent with our *in vitro* functional studies in suggesting that Vorinostat does not impact the frequency of circulating antigen-specific CD8+ T cells.

## Discussion

In this study, we assessed the impact of selected HDACis and PKCms on T cell activation, cytokine production, lytic function, and proliferation. We observed different effects of these drugs in both short- and longer-term assays. Our observations were largely consistent in HIV-1-seropositive and seronegative participants.

Of the drugs tested, a clinically relevant *in vitro* exposure to Vorinostat had the smallest effect on T cells. Relative to mitogenic stimulation with PHA/IL-2, Vorinostat induced very low levels of T cell activation, even when PBMC were continuously exposed to the drug for 24 hours. Vorinostat pre-exposure also had no impact on HIV-specific CD8+ T cell production of lytic molecules, cytokines (with the exception of a modest decrease in TNFα production), or proliferation. Our observations are in agreement with studies that reported Vorinostat impairment of antigen-specific responses in primary cells only occurred following prolonged exposure of cells to Vorinostat or exposure to non-physiological doses^11^,^17^.

Oral Vorinostat dosing also had limited effects on peripheral T cell activation, with the exception of a consistent increase in the frequency of circulating CD25+ T cells. Since Vorinostat did not alter CD25 expression *in vitro*, these changes are more likely associated with previously described effects of Vorinostat on cell migration^32^,^33^,^34^ though we did not investigate this directly. The increased frequencies of CD25+ T cells observed did not impact the circulating frequency of HIV-1-specific CD8+ T cells able to secrete cytokines and degranulate, findings consistent with those of Elliot *et al.*^7^. Together, these data suggest that physiologically relevant exposures to Vorinostat do not alter T cell phenotype or function *ex vivo*.

A physiologically relevant dose and period of exposure of Panobinostat modestly increased CD4+ and CD8+ T cell activation, and induced limited non-specific proliferation in CD8+ T cells, consistent with clinical studies^9^. However, as has previously been reported^17^, Panobinostat consistently reduced antigen-specific CD8+ T cell lytic and proliferative responses.

Like Vorinostat and Panobinostat, Romidepsin induced modest and transient T cell activation. However, while the effects of Vorinostat and Panobinostat on T cell function were largely consistent in short- and longer-term assays, Romidepsin’s effects appeared to depend on assay duration. A 4 hour pre-exposure to Romidepsin did not affect antigen-specific cytokine production by CD8+ T cells measured over the subsequent 6 hours, but significantly reduced antigen-specific proliferation measured over 5 days. We also observed reduced viability in Romidepsin-exposed cultures at 48 and 72 hours but not 24 hours after exposure, consistent with a previous report of ~25% reduction in viability in PBMC that had been exposed to Romidepsin for 48 hours^35^. Similarly, Jones *et al.*’s observations that Romidepsin essentially abrogated antigen-specific IFN-γ production by both primary T cells and clones were made 36 hours after dosing^17^. Together, these data suggest delayed effects of Romidepsin that have implications for T cell function in clinical regimens involving repeated doses of this drug (or Panobinostat). While Søgaard and colleagues did not observe impaired antigen-specific cytokine responses in *ex vivo*-stimulated CD8+ T cells from donors who received multiple doses of Romidepsin^8^, our data suggest that measuring T cell proliferation *ex vivo* will be important to investigate possible longer-term *in vivo* effects of Romidepsin on CD8+ T cell function.

By comparison to the HDACis, the PKCms Prostratin, Ingenol-db and Bryostatin-1 induced significant and sustained activation and robust non-specific cytokine production by T cells. Ingenol-db and Bryostatin-1 also increased non-specific T cell proliferation, and Prostratin and Bryostatin-1 were toxic to PBMC *in vitro*. Recently, Laird *et al.* reported that Bryostatin-1 upregulated CD69 but did not induce toxicity or substantial cytokine production in resting CD4 T cells from HIV-1-uninfected individuals^18^. A recent clinical study also reported that an intravenous Bryostatin-1 infusion did not increase soluble CD14 or IL-6 levels in plasma of cART-suppressed HIV-1-infected participants; however, at the doses used neither PKC activation nor increases in HIV RNA were observed^36^. Moreover, several *in vitro* studies have shown that Bryostatin-1 can promote cytokine production by T cells and myeloid cells^16^,^37^,^38^. Overall, these data suggest that Bryostatin-1 can induce non-specific cytokine production and that these effects are unsurprisingly greater in activated than resting cells.

Ingenol-db and Bryostatin-1 induced non-specific T cell proliferation but also markedly curbed the number of proliferative cycles undergone by antigen-stimulated T cells. In most cell types, PKC activity inhibits proliferation, though the effect may be dependent on the phase of the cell cycle^39^. Ingenol-db and Bryostatin-1 have been described as PKC *agonists* and indeed they initially activate PKC isoforms. However, this activation is followed by rapid degradation, leading to a reduction in cellular PKC levels for up to 24 hours^40^. This means that the short- versus longer-term effects of PKC modulators may be different, which could explain the inability of Bryostatin-1 or Ingenol-db-exposed T cells to divide repeatedly in response to antigen. The sustained reduction of PKC levels following administration of PKC modulators also raises the possibility that these compounds may be unable to induce sustained HIV-1 transcription, as essential host transcription factors such as NFκB are activated via the PKC pathway.

Collectively, our findings raise concerns regarding the safety of incorporating PKC modulators into HIV cure strategies. Bryostatin-1 has been administered to patients undergoing treatment for cancers including metastatic renal cell carcinoma, advanced pancreatic carcinoma, and lymphoma, with reported toxicities including myalgia and lymphopenia^41^,^42^,^43^. More recently, Bryostatin-1 was well tolerated in cART-suppressed HIV-1-infected clinical trial participants, but at doses that did not induce viral reactivation^36^. These findings raise the possibility that efficacious doses of PKCms may be associated with toxicities that are unacceptable in cART-suppressed HIV-1-infected individuals, given their relatively good health and life expectancy^44^. Numerous reports have demonstrated that compounds that induce robust HIV-1 transcription also promote the largest degree of generalized T cell activation, though direct comparisons between viral reactivation and T cell phenotype have not always been made^18^,^36^. Strategies to uncouple these phenomena, allowing the potent latency-reversing capabilities of PKC modulators to be harnessed while minimizing their immunostimulatory properties, should be actively explored. Intriguingly, recent studies have demonstrated that Bryostatin-1 and Prostratin synergized with HDACis *in vitro* to increase HIV-1 mRNA expression in latently-infected cells^18^,^45^,^46^, and such combinations were subsequently shown to reduce the effective concentration of Bryostatin-1^47^. Another approach is the synthesis of new, more potent, PKC modulator derivatives that are less toxic *in vivo* compared to their parent molecule^48^,^49^,^50^, a strategy that is particularly attractive considering the limited availability of naturally-occurring PKC modulators.

Even within the same drug class, different HDACis and PKCms had different effects on T cell phenotype and function. These differences could be due to the different HDAC and PKC isoforms targeted by the compounds, for example, Prostratin can reverse latency by targeting PKCα and θ, whereas Bryostatin-1 mediates reactivation primarily via PKCα and δ^4^,^51^. Additionally, the stability of drug binding to its target may affect the outcome; for example, Vorinostat shows rapid dissociation kinetics, whereas Romidepsin binds its target proteins with much higher affinity^52^. One important difference between compounds that could have implications for the success of “kick and kill” strategies is the timing and duration of effects on antigen-specific CD8+ T cells: Panobinostat and Ingenol-db had rapid effects on T cell function, whereas those of Romidepsin, Prostratin, and Bryostatin-1 were delayed. The variable timing and duration of the effects we observed could explain the inconsistencies between different HIV-1 latency models in terms of whether a particular drug effectively reactivates viral gene expression^53^. Following latency reversal, elimination of reactivated cells by HIV-1-specific CD8+ T cells will only be possible during a “window of opportunity” in which viral antigen is presented on MHC Class I molecules, and any detrimental effects of LRAs on CD8+ T cells during this period could compromise their function. The timing and duration of antigen presentation is currently unclear; however, since we observed effects on antigen-specific CD8+ T cell responses at times ranging from a few hours to five days after dosing, it is likely that the potential effects of LRAs on CD8+ T cell function will need to be considered when designing a latency reversal strategy. The longer-term effects, as well as the sustained T cell activation induced by PKC modulators, may also have implications for repeated dosing. While none of the LRAs tested induced sustained downmodulation of MHC Class I on CD4 T cells nor completely abrogated antigen-specific CD8+ T cell responses in our assays, a critical question is whether the detrimental effects we observed *in vitro* would be sufficient to compromise clearance of infected cells *in vivo*. One way to address this question would be to test the ability of CD8+ T cells from clinical trial participants to kill HIV-1-infected cells in viral inhibition assays or latency clearance assays *ex vivo* before and after dosing^11^.

Our study has some limitations. Given the technical challenges associated with measuring PKC modulator pharmacokinetics *in vivo*^27^, more work is needed to determine *in vitro* doses of these compounds that reflect efficacious clinically-achievable exposures, particularly as phenotypic and functional outcomes may be highly dose-dependent^36^,^54^,^55^. The increased expression of CD25 on T cells after an oral dose of Vorinostat but not following exposure to the drug *in vitro* suggests that LRAs may have effects on T cell tissue distribution that could not be measured in a static *in vitro* system. We did not directly assess the impact of LRAs on immune cells such as macrophages that may play a role in the maintenance of the viral reservoir^56^,^57^ and modulating other immune cells, including T cells. Our observation that Bryostatin-1 induced expression of IL-12 (p70), which is largely produced by myeloid cells, suggests possible modulation of these cells by LRAs, though we cannot exclude the possibility that this effect may have been mediated indirectly, via IFN-γ produced by T cells. We were unable to model the effect of repeated doses *in vitro* but will seek to address this issue in future studies using *ex vivo* samples collected from a multi-dose Vorinostat trial^58^.

These data advance on previous work in a number of ways. To our knowledge, we have conducted the first comprehensive analysis of the effects of two major classes of latency reversing agents on T cell activation, cytokine production and proliferation in PBMC from HIV-1-infected individuals on cART. We have demonstrated the importance of timing when considering whether these compounds impact antigen-specific CD8+ T cell responses, providing data that help to reconcile divergent *in vitro* and *in vivo* reports^7^,^8^,^17^. By focusing on physiologically relevant doses *and* times of exposure to evaluate the effects of HDACis, our data indicate that the detrimental effects of these drugs on CD8+ T cells may be more modest than previously suggested. Finally, we report robust non-specific effects of PKC modulators on T cells, highlighting the need to tailor regimens involving these drugs to minimize the risk of toxicity *in vivo*.

## Methods

### Study subjects

HIV-1-infected participants were recruited from the UNC HIV Clinical Trials Unit. Participants (all male; age range 25–65) were receiving stable standard-of-care antiretroviral therapy (range 2–21 years) and had maintained plasma HIV-1 RNA < 50 copies/ml and a CD4 T cell count of >300/μl for ≥6 months before enrolment. Participants receiving oral Vorinostat (donated by Merck Research Laboratories, West Point, PA) were administered a 400 mg dose. HIV-1-seronegative participants were recruited by the UNC CFAR Immunology Core.

### Ethics Statement

All participants provided written informed consent. All experimental protocols were approved by the University of North Carolina Institutional Biomedical Review Board (ethics numbers: 14–0741, 11–0228, and 13–3613) and carried out in accordance with the relevant guidelines.

### Peripheral blood mononuclear cell and plasma isolation

Blood was collected by venipuncture. Peripheral blood mononuclear cells (PBMC) and plasma were isolated by density gradient centrifugation and frozen within 6 hours of sampling. In selected experiments, leukocytes were obtained from HIV-1-seropositive donors by continuous-flow leukapheresis. Unless otherwise stated, PBMC were frozen then stored in liquid nitrogen prior to use. Plasma was stored at −80 °C.

### Putative latency-reversing agents (LRAs)

Vorinostat (Merck), Romidepsin and Panobinostat (both Selleckchem), Prostratin (LC Laboratories), Ingenol 3,20-dibenzoate (Santa Cruz Biotechnology), and Bryostatin-1 (NCI) were obtained through the Martin Delaney Collaboratory of AIDS Researchers for Eradication (CARE) Pharmacology Core. All compounds were solubilized in DMSO, except Bryostatin-1, which was solubilized in 100% Ethanol. The final drug concentration in all *ex vivo* experiments was 335 nM for Vorinostat, 20 nM for Romidepsin, 20 nM for Panobinostat, 1 μM for Prostratin, 100 nM for Ingenol 3,20-dibenzoate, and 25 nM for Brostatin-1.

### T cell activation experiments

Cryopreserved PBMC were thawed and rested at 37 °C overnight in R10 (RPMI 1640 medium supplemented with 10% fetal bovine serum; Penicillin/streptomycin; 2 nM L-glutamine; 1 nM sodium pyruvate and 10 mM HEPES). PBMC were cultured in the presence of LRAs at 37 °C. Drug was removed by washing PBMC twice with R10. R10 + 0.5% DMSO was used as the vehicle control. The positive control was 3 μg/mL PHA + 60 units/mL IL-2. Supernatants were harvested for cytokine analysis (see below). PBMC were stained with Zombie NIR viability dye, then CD3-PE-Dazzle 594; CD4-Alexa fluor 488; CD8-Brilliant Violet 510; PerCP-conjugated CD14, 16, 19, and 56 (dump channel); CD25-PE; CD38-PE-Cy7; CD45RO-Brilliant Violet 650; CD69-APC; HLA-DR-Alexa-fluor 700; MHC Class I-Pacific blue (clone W6/32); and PD-1-Brilliant Violet 605 (all Biolegend). Cells were acquired on an LSRII flow cytometer (BD Biosciences) and analyzed using FlowJo version 10 (Tree Star). Gates for positive events were positioned using fluorescence minus one (FMO) controls (Supplementary Fig. S1). Due to the previously reported downmodulation of CD4 by the PKCms Bryostatin-1 and Prostratin^4^,^59^, in assays where these compounds were used CD3+ CD8- lymphocytes were considered CD4+ T cells.

### Measurement of LRA concentrations

Samples were extracted by protein precipitation with acetonitrile containing internal standard compounds and mixed with water prior to LC-MS/MS analysis. A Waters Atlantis T3 reverse phase analytical column was used to separate matrix components from method compounds with detection on an AB Sciex API-5000 triple quadrupole mass spectrometer. Calibration standards and QC samples were prepared in RPMI/10% FBS. The analytical range was 1–1000 ng/mL for Vorinostat, Panobinostat, Prostratin, and Ingenol 3,20-dibenzoate, 0.71–710 ng/mL for Romidepsin, and 5–10 000 ng/mL for Bryostatin-1. Internal standard compounds were Nevirapine-d_3_ for Vorinostat, Zidovudine-d_4_ for Panobinostat, Raltegravir-d^3^ for Romidepsin and Prostratin, Ritonavir-d6 for Ingenol 3,20-dibenzoate, and Lopinavir-d8 for Bryostatin-1. Vorinostat concentrations in human serum were measured as previously^6^ at 0.5, 1, 3, and 10 hours after dosing.

### Luminex assay

Cytokines and chemokines were measured in undiluted cell culture supernatants using the Bio-Plex luminex assay (Bio-Rad) and the MSD Multi-spot Assay system (MSD) according to the manufacturers’ instructions. Data were acquired using a Bio-Plex^®^ MAGPIX^TM^ Multiplex Reader (Bio-Rad).

### Peptides

Peptides were synthesized by Sigma Genosys. HIV-1 CD8+ optimal epitopes (http://www.hiv.lanl.gov/content/sequence/ELF/epitope_analyzer.html), grouped by protein (either Gag/Nef or non-Gag/NEF HIV-1 proteins), were used to measure antigen-specific responses in HIV-1-seropositive participants. A pool of ‘Flu, EBV, and CMV CD8+ epitopes^60^ was used to measure antigen-specific responses in both HIV-1-seropositive and -seronegative donors.

### Intracellular cytokine staining

Cryopreserved PBMC were thawed and rested at 37 °C overnight in R10, then exposed to LRAs for 4 hours. Due to sample availability, not all LRAs were tested in all participants. R10 + 0.5% DMSO was the vehicle control. Drug was removed by washing twice with R10, and PBMC were incubated with peptides (2 μg/mL) for 6 hours at 37 °C in the presence of monensin and CD107a-APC (Biolegend). PBMC from HIV-1-seropositive participants were exposed to HIV-1 CD8+ optimal epitopes, and PBMC from HIV-1-seronegative participants were exposed to a pool of ‘Flu, EBV, and CMV (FEC) CD8+ epitopes. R10 + 0.5% DMSO was the no-peptide control. PBMC were then stained with Zombie NIR viability dye, followed by CD3-PE-Dazzle 594; CD4-Alexa fluor 488; CD8-Brilliant Violet 510; PerCP-conjugated CD14, 16, 19, and 56 (dump channel); CD45RA-Brilliant Violet 650; and CCR7-Brilliant Violet 605 (all Biolegend). They were fixed, permeabilized and stained intracellularly with IFN-γ-PE; Perforin-Brilliant Violet 421 (clone B-D48); TNFα-Alexa fluor 700 (all Biolegend); and MIP-1β-PE-Cy7 (BD Biosciences). Cells were acquired using an LSRII flow cytometer as previously. A minimum of 5000 viable CD8+ events were collected. Responses were expressed as frequency of non-naïve CD8+ T cells (CD45RA+ CCR7+ cells were considered naïve). Data were included in subsequent analyses if the antigen-specific response of cells pre-exposed to vehicle control met the following criteria: above mean background (vehicle control followed by no-peptide control) +2 standard deviations, and above a threshold of 0.1%.

### T cell proliferation assay

Cryopreserved PBMC were thawed and rested at 37 °C overnight in R10, then pulsed under rotation with 5 μM carboxyfluorescein succinimidyl ester (CFSE) for 10 minutes. The reaction was quenched with ice cold R10 and excess CFSE removed by washing twice with PBS + 5% FBS. PBMC were then exposed to LRA or vehicle (R10 + 0.5% DMSO) for 4 hours, washed and incubated with 2 μg/mL peptide + 20 units/mL IL-2 at 37 °C for 5 days. R10 + 0.5% DMSO + 20 units/mL was the vehicle control and 3 μg/ml PHA + 20 units/mL IL-2 was the positive control. After 5 days, the PBMC were stained with Zombie NIR viability dye, followed by CD3-PE-Dazzle 594; CD4-Brilliant Violet 650 and CD8-Brilliant Violet 510, then acquired using an LSRII flow cytometer as previously. Data were analyzed using FlowJo version 10 and ModFitLT version 4 (Verity Software House).

### Measurement of resting CD4+ T cell-associated RNA

Resting CD4+ T cell-associated HIV RNA was quantified as previously^6^. Total RNA was isolated from 36 pools of 10^6^ resting cells at each time point.

### Statistical analyses

Outcomes were compared between paired drug and vehicle samples using an exact Wilcoxon Signed Rank test (SAS), and analyses including both HIV-1-seropositive and HIV-seronegative participants used an exact Wilcoxon Signed Rank test stratified by HIV-1 status (StatXact version 10). Unpaired measurements of resting CD4+ T cell-associated HIV-1 RNA at baseline and after an oral dose of Vorinostat (Fig. 7) were compared using a Mann-Whitney test (Graphpad Prism Version 6). All p-values are 2-sided and were not adjusted for multiple comparisons; unadjusted p < 0.05 are displayed in the figures. Where a single measurement is discussed in the text, an exact p-value is given. Where multiple measurements are discussed together, an approximate p-value (e.g. p < 0.05) is given.

In this study unadjusted p-values 0.016 and below remained significant at the 0.05 level with false discovery rate (FDR) adjustment accounting for the 2336 statistical tests conducted. In all figures, p-values < 0.05 are indicated by one asterisk (*) and p-values ≤ 0.016 are indicated by two asterisks (**).

## Additional Information

**How to cite this article**: Clutton, G. *et al.* The differential short- and long-term effects of HIV-1 latency reversing agents on T cell function. *Sci. Rep.* **6**, 30749; doi: 10.1038/srep30749 (2016).

## Supplementary Material

Supplementary Information

## Figures and Tables

**Table 1:** LRAs were removed from cell culture following washing.

Supernatant concentrations at 24 hours (nM)^a^												
Time in drug (hours)	Vorinostat		Romidepsin		Panobinostat		Prostratin		Ingenol-db		Bryostatin-1	
	Mean	CV	Mean	CV	Mean	CV	Mean	CV	Mean	CV	Mean	CV
3	<3.8	N/A	<1.3	N/A	<2.9	N/A	<2.6	N/A	6.25	0.10	5.78	0.07
6	<3.8	N/A	<1.3	N/A	<2.9	N/A	<2.6	N/A	4.68	0.06	<5.5	N/A
12	<3.8	N/A	<1.3	N/A	<2.9	N/A	<2.6	N/A	4.92	0.20	<5.5	N/A
24	213	0.09	18.7	0.06	7.6	0.07	1099	0.04	97.2	0.04	25.7	0.04

^a^Measured in triplicate by LC-MS/MS. N/A: not applicable.

**Table 2:** Effects of pre-exposure^a^ to LRAs on antigen-specific CD8+ T cell function^b^.

Function		335 nM Vorinostat	20 nM Romidepsin	20 nM Panobinostat	1 μM Prostratin	100 nM Ingenol-db	10 nM Bryostatin-1
IFN-γ+	n (HIV+)^c^	10 (5)	10 (5)	**10 (5)**	10 (5)	**10 (5)**	**10 (5)**
	Fold-change	0.89	0.91	**0.68**	1.02	**2.00**	**1.51**
	CV	0.16	0.29	**0.24**	0.42	**0.37**	**0.53**
	p-value^d^	0.107	0.926	**0.002**	0.647	**0.041**	**0.047**
TNFα+	n (HIV+)	**6 (2)**	6 (2)	6 (2)	6 (2)	**6 (2)**	6 (2)
	Fold-change	**0.67**	0.76	0.49	1.17	**2.77**	2.27
	CV	**0.42**	0.55	0.82	0.35	**0.59**	1.02
	p-value	**0.031**	0.344	0.094	0.344	**0.031**	0.344
IFN-γ+ TNFα+	n (HIV+)	**7 (3)**	7 (3)	**7 (3)**	8 (3)	7 (3)	**8 (3)**
	Fold-change	**0.65**	0.74	**0.57**	1.35	2.45	**1.91**
	CV	**0.34**	0.55	**0.56**	0.71	0.52	**0.41**
	p-value	**0.016**	0.672	**0.016**	0.648	0.094	**0.023**
IFN-γ+ TNFα+ MIP-1β+ CD107a+	n	**7 (3)**	7 (3)	**7 (3)**	6 (3)	7 (3)	7 (3)
	Fold-change	**0.67**	0.80	**0.57**	1.00	2.22	0.82
	CV	**0.31**	0.55	**0.53**	0.23	0.52	0.76
	p-value	**0.016**	0.469	**0.016**	0.844	0.094	1.000

^a^PBMC were exposed to LRA for 4 hours, washed and then stimulated with peptides for 6 hours.

^b^Gated on non-naïve (naïve = CD45RA + CCR7+) CD8 + T cells.

^c^Total n, with number of HIV-1-seropositive participants in brackets. For each function, data were included only if the antigen-specific response met the criteria for positivity as defined in the Methods. The number of participants who met these criteria differed between different functions.

^d^Measurements were compared between vehicle and LRA treatment using an exact Stratified Wilcoxon Signed Rank test.
