# Corrigendum: The differential short- and long-term effects of HIV-1 latency-reversing agents on T cell function

**DOI:** 10.1038/srep34430

**Published:** 2016-10-03

**Authors:** G. Clutton, Y. Xu, P. L. Baldoni, K. R. Mollan, J. Kirchherr, W. Newhard, Kara Cox, J. D. Kuruc, A. Kashuba, R. Barnard, N. Archin, C. L. Gay, M. G. Hudgens, D. M. Margolis, N. Goonetilleke

Scientific Reports 6: Article number: 3074910.1038/srep30749; published online: 08 02 2016; updated: 10 03 2016

The Acknowledgements section in this Article is incomplete.

“We thank all the study participants. For technical assistance we thank Kuo Yang and members of the UNC Clinical Trials Unit, the UNC CFAR Immunology Core and the UNC Flow Cytometry Core Facility. The UNC Flow Cytometry Core Facility is supported in part by P30 CA016086 Cancer Center Core Support Grant to the UNC Lineberger Comprehensive Cancer Center”.

should read:

“We thank all the study participants. For technical assistance we thank Kuo Yang and members of the UNC Clinical Trials Unit, the UNC CFAR Immunology Core and the UNC Flow Cytometry Core Facility. This study was supported by NIH grants to the Collaboratory of AIDS Researchers for Eradication (CARE; U19 AI096113) and the UNC Center for AIDS Research (CFAR; P30 AI50410). This research was also supported by the Creative and Novel Ideas in HIV Research Program (CNIHR) through a supplement to the University of California at San Francisco (UCSF) Center For AIDS Research funding (P30 AI027763). This funding was made possible by collaborative efforts of the Office of AIDS Research, the National Institute of Allergy and Infectious Diseases, and the International AIDS Society. The UNC Flow Cytometry Core Facility is supported in part by P30 CA016086 Cancer Center Core Support Grant to the UNC Lineberger Comprehensive Cancer Center”.

