# Supplementary material for: The differential short- and long-term effects of HIV-1 latency-reversing agents on T cell function
Source: Sci Rep. 2016 Aug 2;6:30749. doi: 10.1038/srep30749 (PMC4969750; doi:10.1038/srep30749)

Supplementary Information

The differential short- and long-term effects of HIV-1 latency -reversing agents on T cell function

G Clutton[1], Y Xu[1], PL Baldoni[2], KR Mollan[3], J Kirchherr[4], W Newhard[5], Kara Cox[5], JD Kuruc[4], A Kashuba[6], R Barnard[5], N Archin[4], CL Gay[4], MG Hudgens[2], DM Margolis[1,4], N Goonetilleke[1,4]

[1] Department of Microbiology & Immunology, UNC Chapel Hill School of Medicine, Chapel Hill, North Carolina, USA

[2] Department of Biostatistics, UNC Chapel Hill, Chapel Hill, North Carolina, USA

[3] Lineberger Comprehensive Care Center, UNC Chapel Hill, Chapel Hill, North Carolina, USA

[4] Department of Medicine and UNC HIV Cure Center, UNC Chapel Hill School of Medicine, Chapel Hill, North Carolina, USA

[5]Merck Research Laboratories, White Horse Junction, Pennsylvania, USA

[6] Eshelman School of Pharmacy, UNC Chapel Hill, North Carolina, USA

Titles and Legends for Supplementary Figures

Supplementary Figure S1. Gating strategy for activation markers on (a) CD4+ and (b) CD8+ T cells. Expression of T cell activation markers was assessed by flow cytometry. Gates for positive events were set using fluorescence minus one (FMO) controls. For the gating strategy to identify CD4+ and CD8+ T cells, see Supplementary Fig S9.

Supplementary Figure S2. Expression of activation markers on Ingenol-db- or mitogen-stimulated T cells. Expression of activation markers on CD4+ and CD8+ T cells exposed to Ingenol-db or PHA/IL-2. PBMC from HIV-seropositive participants durably suppressed on cART (n=7) were exposed to vehicle (0.5% DMSO), Ingenol-db, or PHA/IL-2 for 3, 6, 12, or 24 hours, washed to remove extracellular drug, and maintained in vehicle for the remainder of the culture period. Activation marker expression was assessed at 24 hours. MFI, mean fluorescence intensity. Measurements were compared between vehicle and Ingenol-db or PHA/IL-2 treatment using an exact Wilcoxon Signed Rank test. * $p < 0.05$; ** $p \leq 0.016$.

Supplementary Figure S3. Expression of activation markers on memory T cells exposed to HDACis. PBMC from HIV-seropositive participants durably suppressed on cART (n=7) were exposed to vehicle (0.5% DMSO) or HDACis for 3, 6, 12, or 24 hours, washed to remove extracellular drug, and maintained in vehicle for the remainder of the culture period. Activation and memory marker expression was measured at 24 hours. (A) Expression of the T cell memory marker CD45RO on CD4+ and CD8+ T cells. (B) Expression of activation markers on memory (CD45RO+) CD4+ and CD8+ T cells. MFI, mean fluorescence intensity. Measurements were compared between vehicle and HDACi treatment using an exact Wilcoxon Signed Rank test. * $p < 0.05$; ** $p \leq 0.016$.

Supplementary Figure S4. Expression of activation markers on T cells exposed to HDACis or PKCms. PBMC from HIV-seronegative participants (n=7) were exposed to vehicle (0.5% DMSO) or LRA for 3 hours, washed to remove extracellular drug, and maintained in vehicle for the remainder of the culture period. Activation marker expression was measured at 24, 48, and 72 hours. MFI, mean fluorescence intensity.

Supplementary Figure S5. Expression of activation markers over 72 hours on mitogen-stimulated T cells. Expression of activation markers on CD4+ and CD8+ T cells exposed to the positive control PHA/IL-2. PBMC from HIV-seropositive participants durably suppressed on cART (n=8) were exposed to vehicle (0.5% DMSO) or PHA/IL-2 for 3 hours, washed to remove extracellular drug, and maintained in vehicle for 72 hours. Activation marker expression was assessed at 24, 48, and 72 hours. Measurements were compared between vehicle and PHA/IL-2 treatment using an exact Wilcoxon Signed Rank test. * $p < 0.05$; ** $p \leq 0.016$.

Supplementary Figure S6. Expression of activation markers on memory T cells exposed to HDACis or PKCms. PBMC from HIV-seropositive participants durably suppressed on cART (n=7) were exposed to vehicle (0.5% DMSO) or LRA for 3 hours, washed to remove extracellular drug, and maintained in vehicle

for the remainder of the culture period. Activation and memory marker expression was measured at 24, 48, and 72 hours. (a) Expression of the T cell memory marker CD45RO on CD4+ and CD8+ T cells. (b) Expression of activation markers on memory (CD45RO+) CD4+ and CD8+ T cells. MFI, mean fluorescence intensity. Measurements were compared between vehicle and LRA treatment using an exact Wilcoxon Signed Rank test. * $p < 0.05$; ** $p \leq 0.016$.

Supplementary Figure S7. Expression of activation markers on T cells exposed to HDACis or PKCms for 6 hours. PBMC from HIV-seropositive participants durably suppressed on cART (n=8) were exposed to vehicle (0.5% DMSO) or LRA for 6 hours, washed to remove extracellular drug, and maintained in vehicle for the remainder of the culture period. Activation marker expression was measured at 24, 48, and 72 hours. MFI, mean fluorescence intensity. Measurements were compared between vehicle and LRA-treatment using an exact Wilcoxon Signed Rank test. * $p < 0.05$; ** $p \leq 0.016$.

Supplementary Figure S8. HDACis do not induce non-specific cytokine release. PBMC from HIV-seropositive participants durably suppressed on cART (n=5) were exposed to vehicle (0.5% DMSO) or HDACis for 3 hours, washed to remove extracellular drug, and maintained in vehicle for the remainder of the culture period. Cytokine concentrations were measured in supernatants collected at 24 hours. Measurements were compared between vehicle and HDACi treatment using an exact Wilcoxon Signed Rank test

Supplementary Figure S9. Cytokine concentrations in culture supernatants following exposure to LRAs. PBMC from HIV-seropositive participants durably suppressed on cART (n=5-8, depending on sample availability) were exposed to vehicle (0.5% DMSO) or LRAs for (a) 3 or (b) 6 hours, washed to remove extracellular drug, and maintained in vehicle for the remainder of the culture period. Cytokine concentrations were measured in supernatants collected at 24, 48, and 72 hours. Measurements were compared between vehicle and LRA-treatment using an exact Wilcoxon Signed Rank test. * $p < 0.05$; ** $p \leq 0.016$.

Supplementary Figure S10. Cytokine and lytic granule release by CD8+ T cells. Expression of cytokines and lytic molecules by T cells was assessed by flow cytometry. Gating strategy for the identification of CD8+ T cells expressing cytokines and lytic molecules in response to vehicle (middle row) or FEC peptide (bottom row) stimulation.

Supplementary Figure S11. Effects of an oral dose of Vorinostat on T cell phenotype *ex vivo*. Participants (n=3) received a single 400 mg oral dose of Vorinostat. Phenotype was assessed by flow cytometry on PBMC collected immediately prior to dosing and 4, 7, 10, and 24 hours post-dose. (a) Expression of MHC Class I on peripheral blood CD4+ and CD8+ T cells. MFI, mean fluorescence intensity. (b) Frequency of naïve (CD45RA+ CCR7+), central memory (TCM) (CD45RA- CCR7+), effector memory (TEM) (CD45RA- CCR7-), terminally differentiated (TEMRA) (CD45RA+ CCR7-), and resting (CD25- CD38- CD69- HLA-DR-) CD4+ T cells.

Supplementary Figure S12. Effects of an oral dose of Vorinostat on memory T cell phenotype and function *ex vivo*. Participants (n=3) received a single 400 mg oral dose of Vorinostat. (a) Expression of the T cell memory marker CD45RO on peripheral blood CD4+ and CD8+ T cells immediately prior to

dosing and 4, 7, 10, and 24 hours post-dose. (b) Expression of activation markers on peripheral blood memory (CD45RO+) CD4+ and CD8+ T cells immediately prior to dosing and 4, 7, 10, and 24 hours post-dose. MFI, mean fluorescence intensity.

Supplementary Figure S1

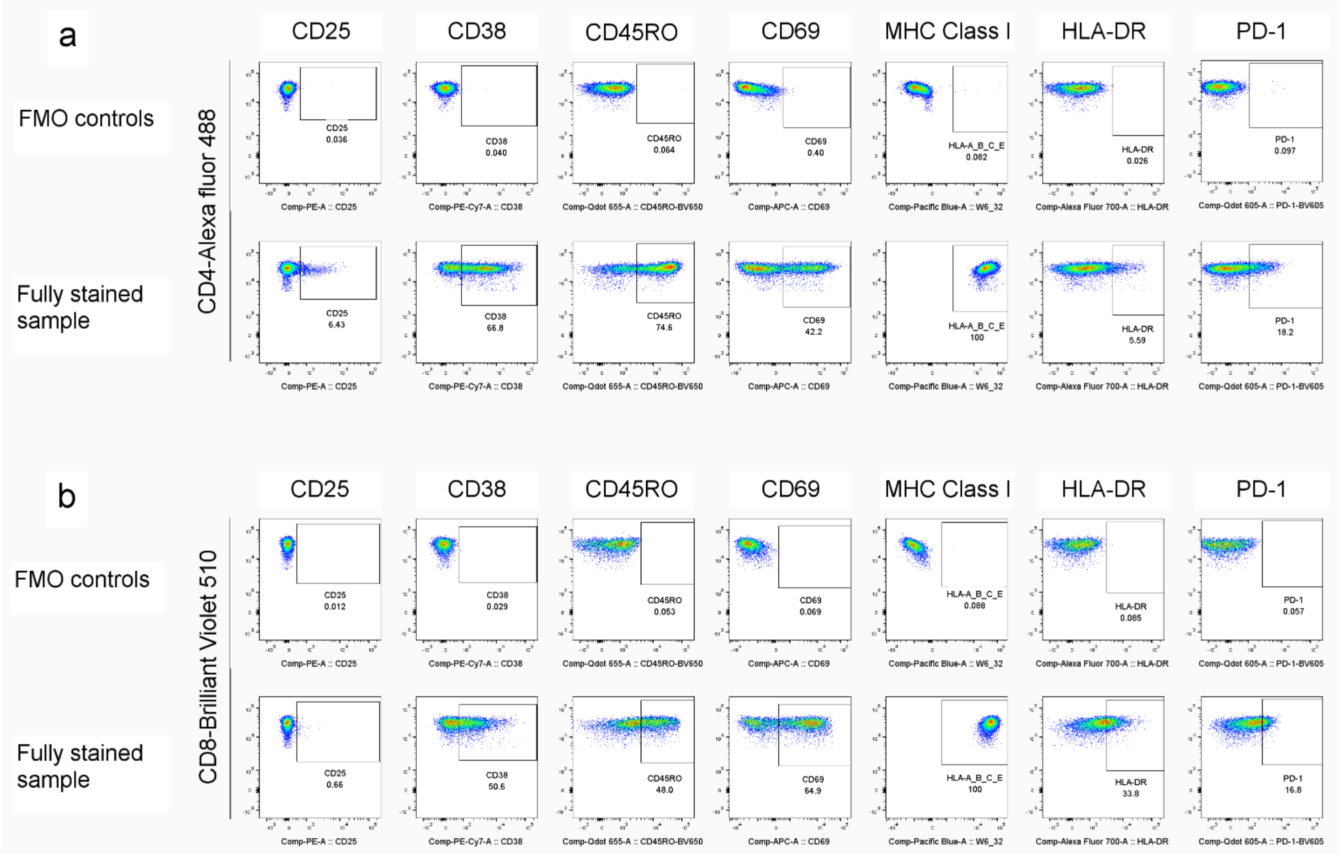

Supplementary Figure S2

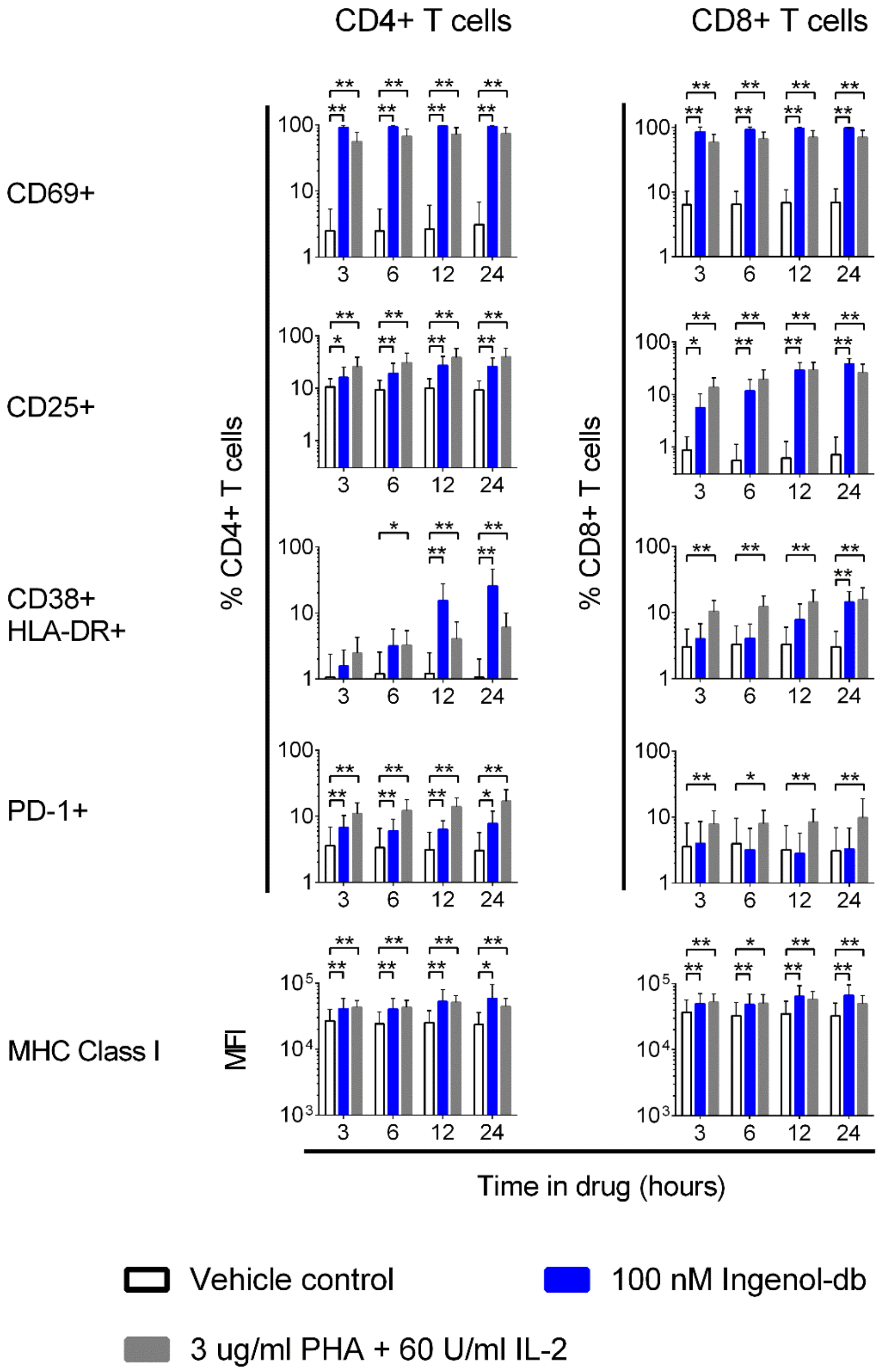

Supplementary Figure S3

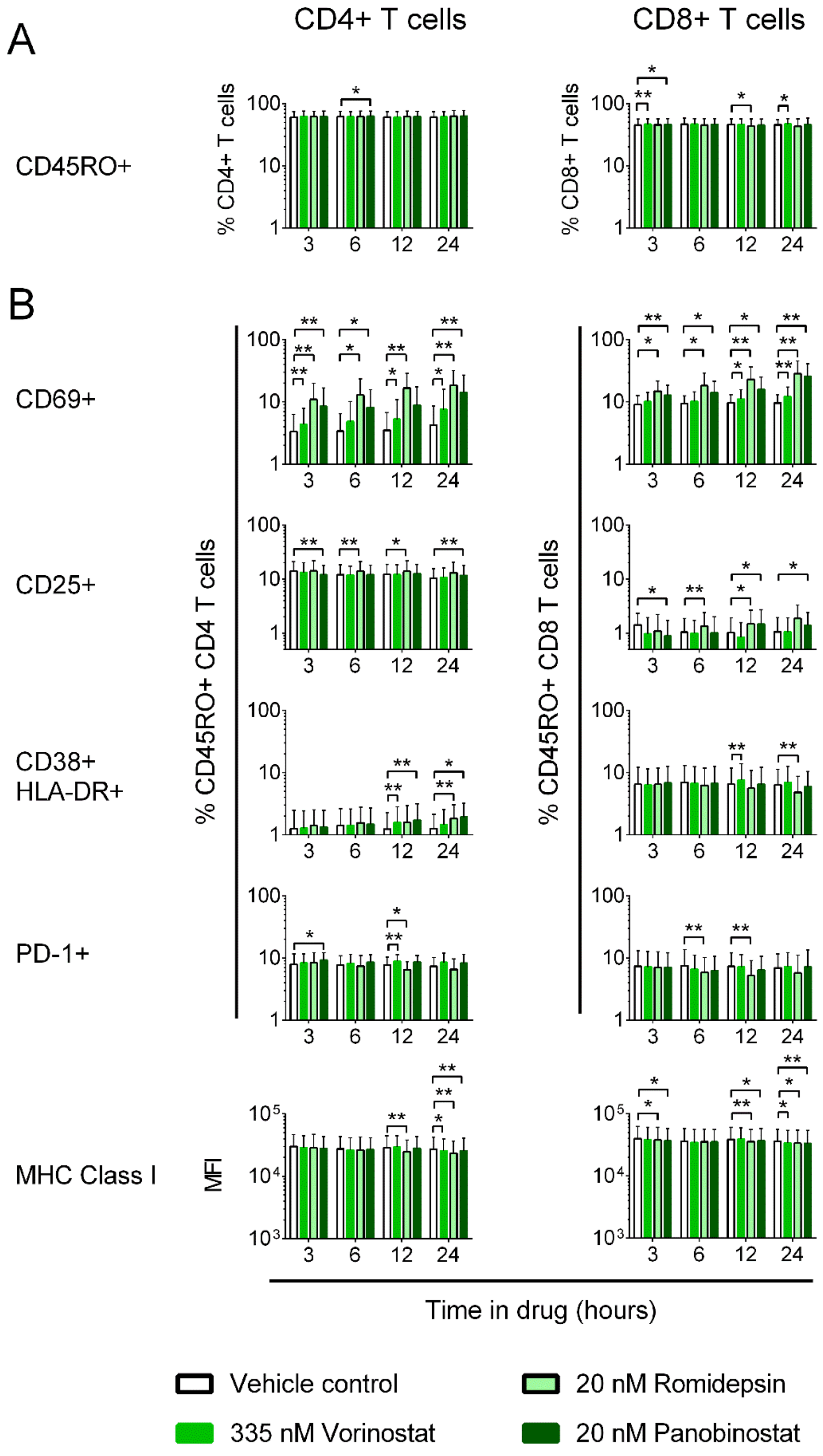

Supplementary Figure S4

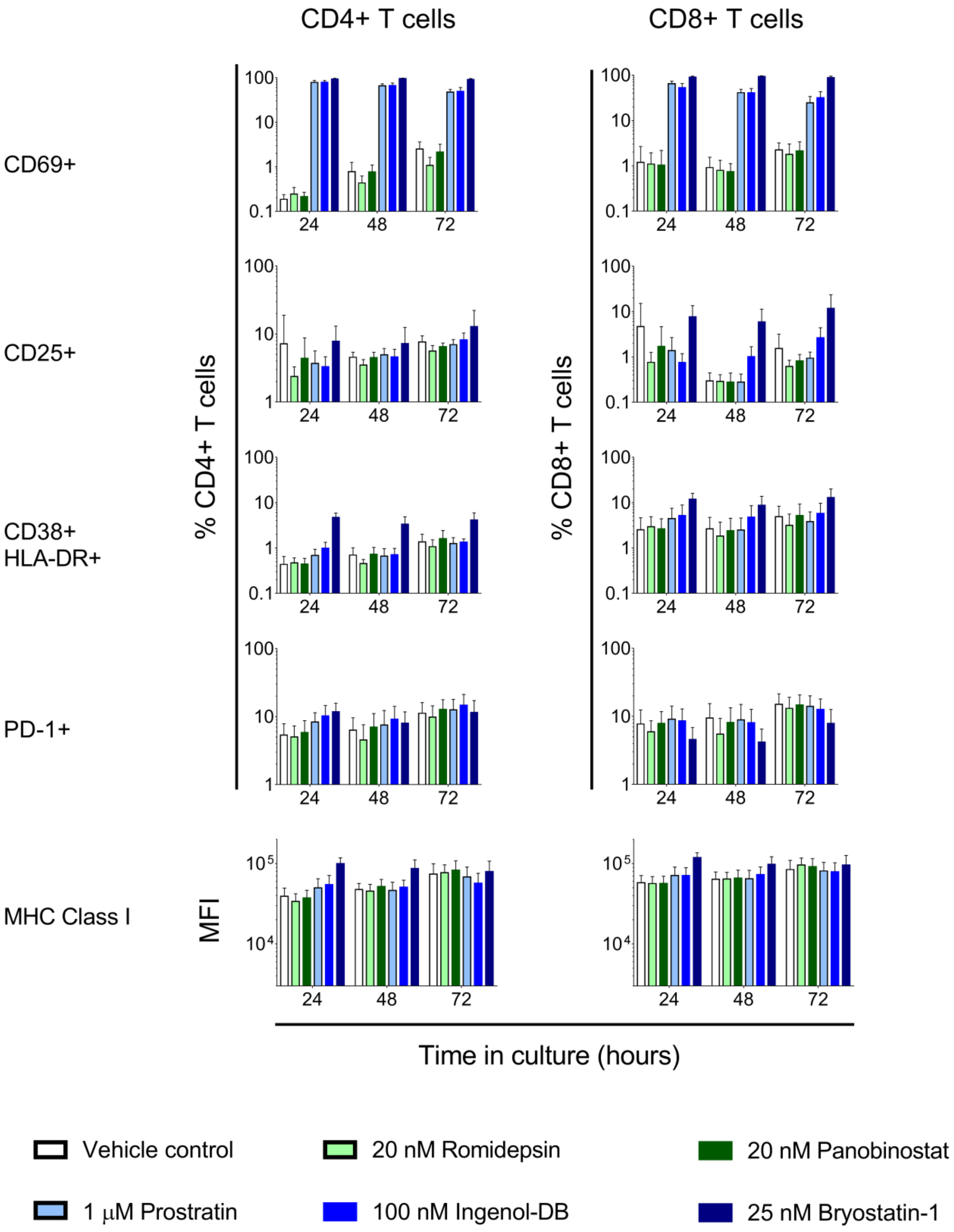

Supplementary Figure S5

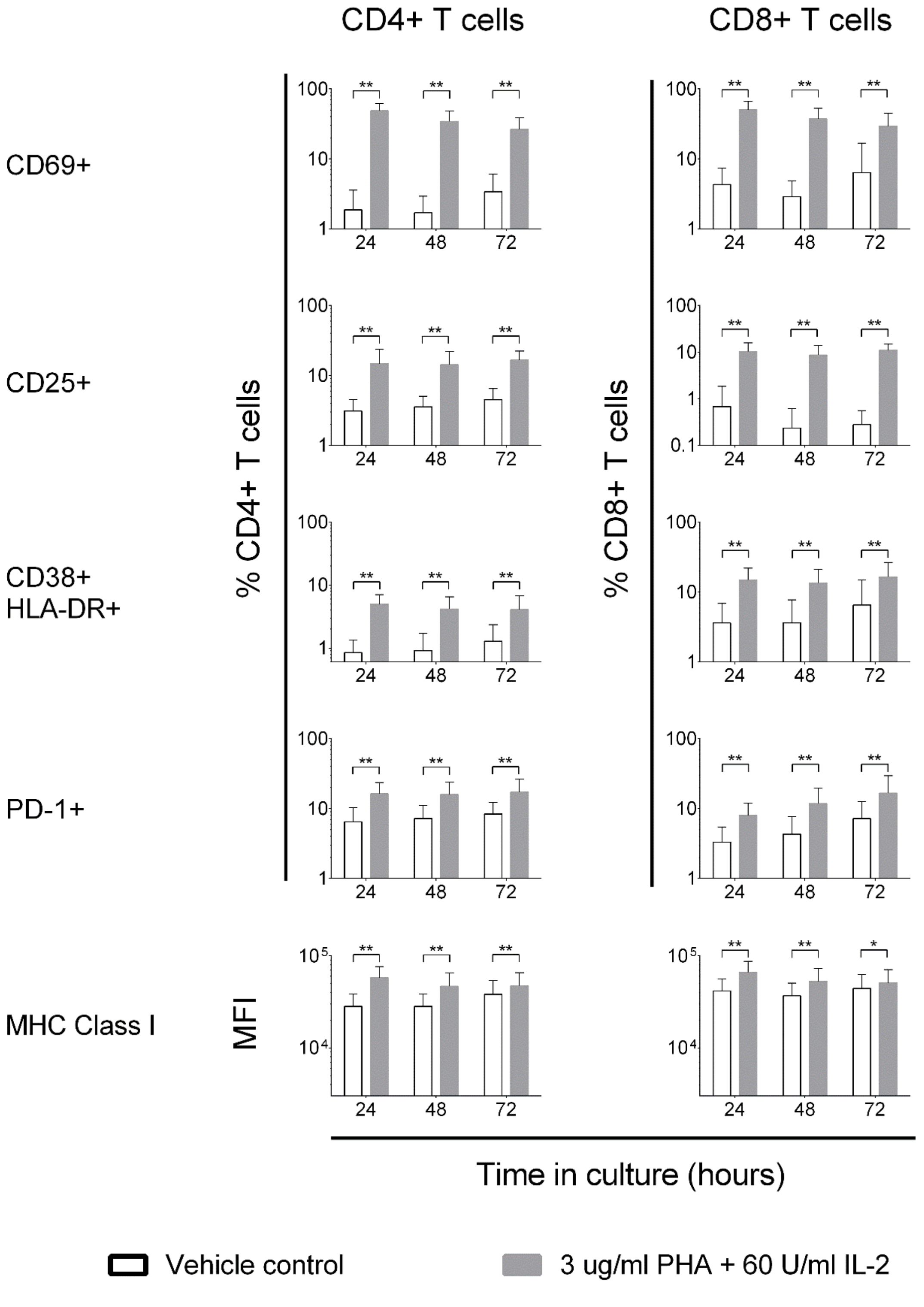

Supplementary Figure S6

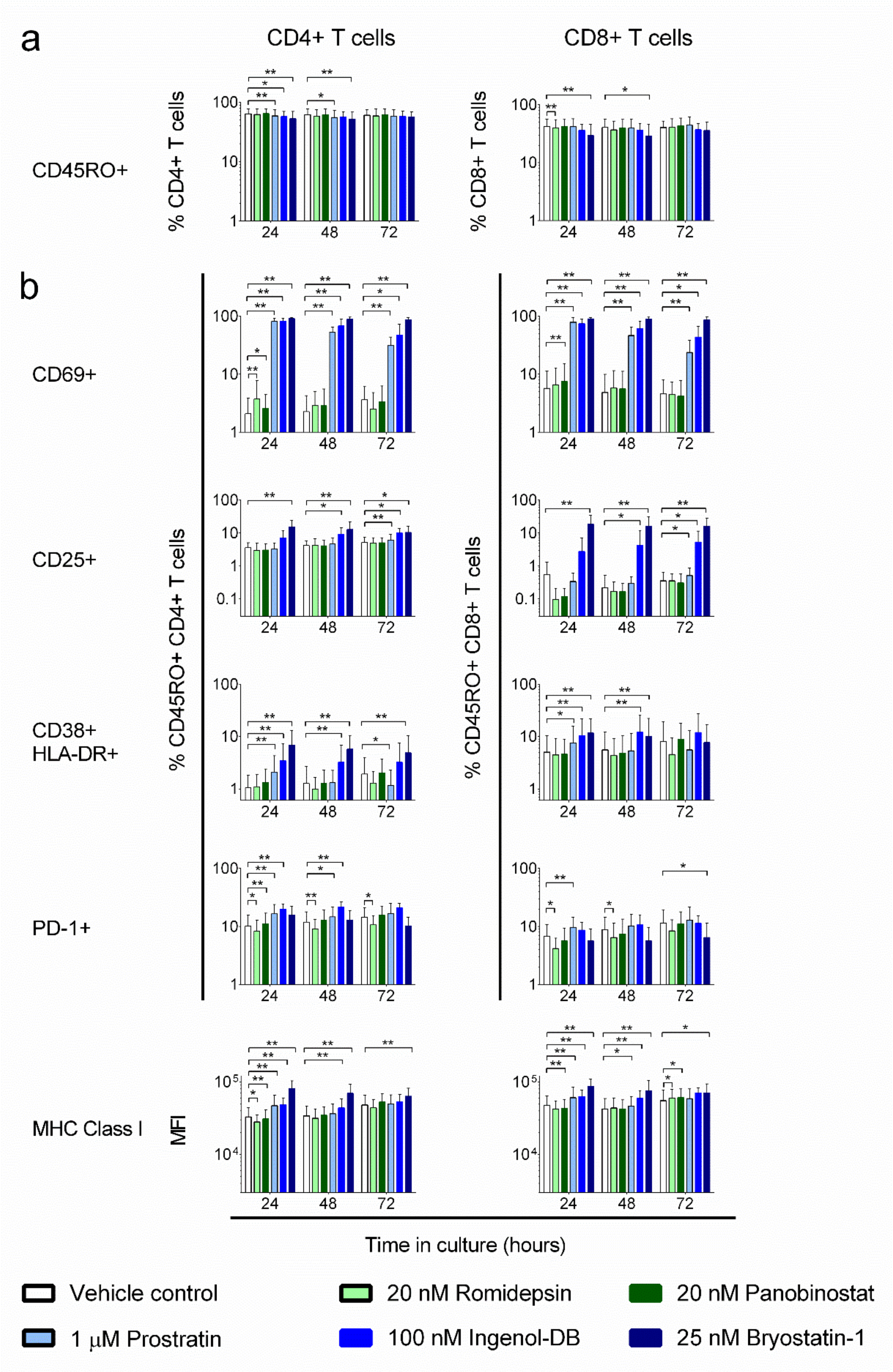

Supplementary Figure S7

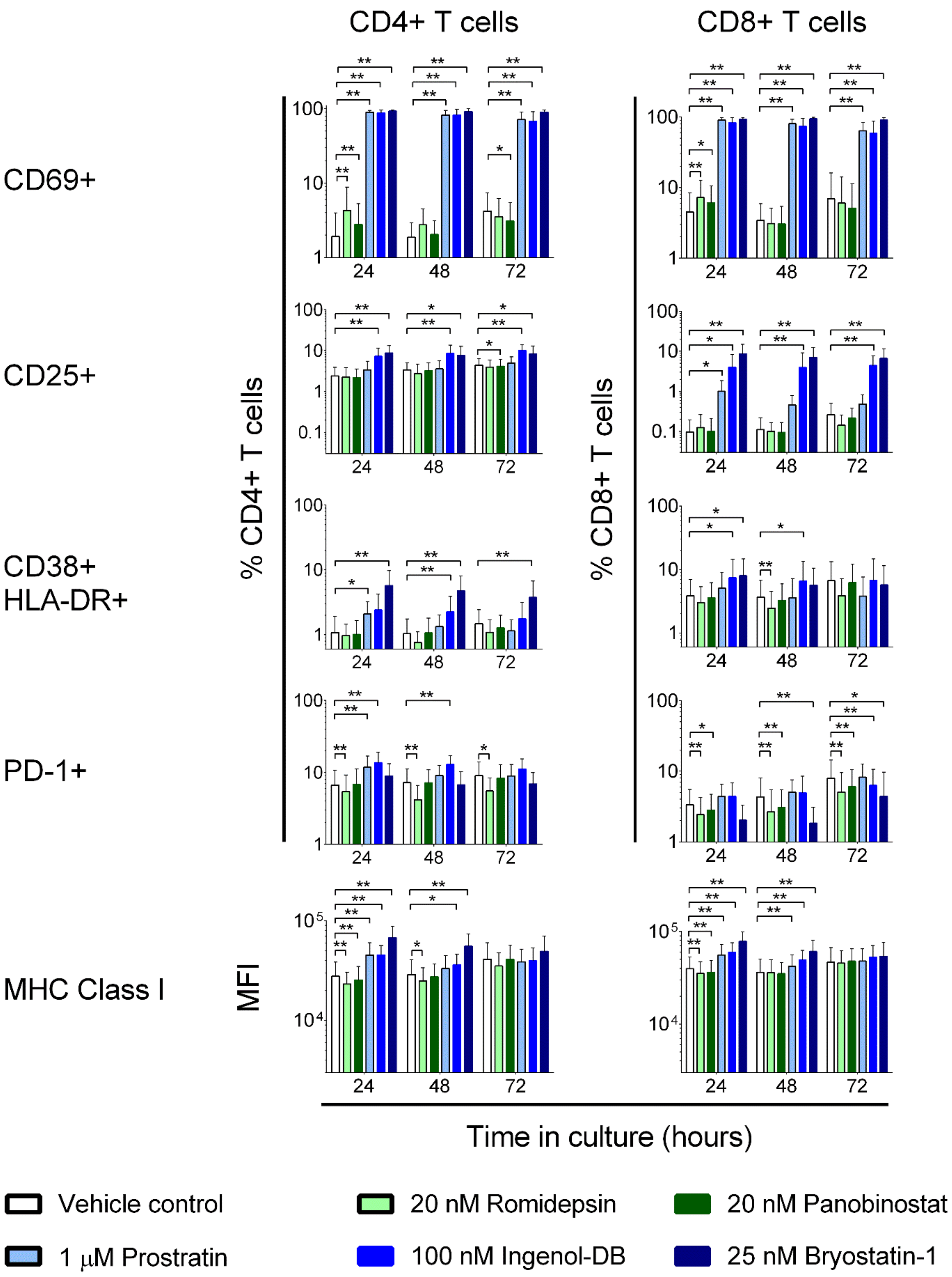

Supplementary Figure S8

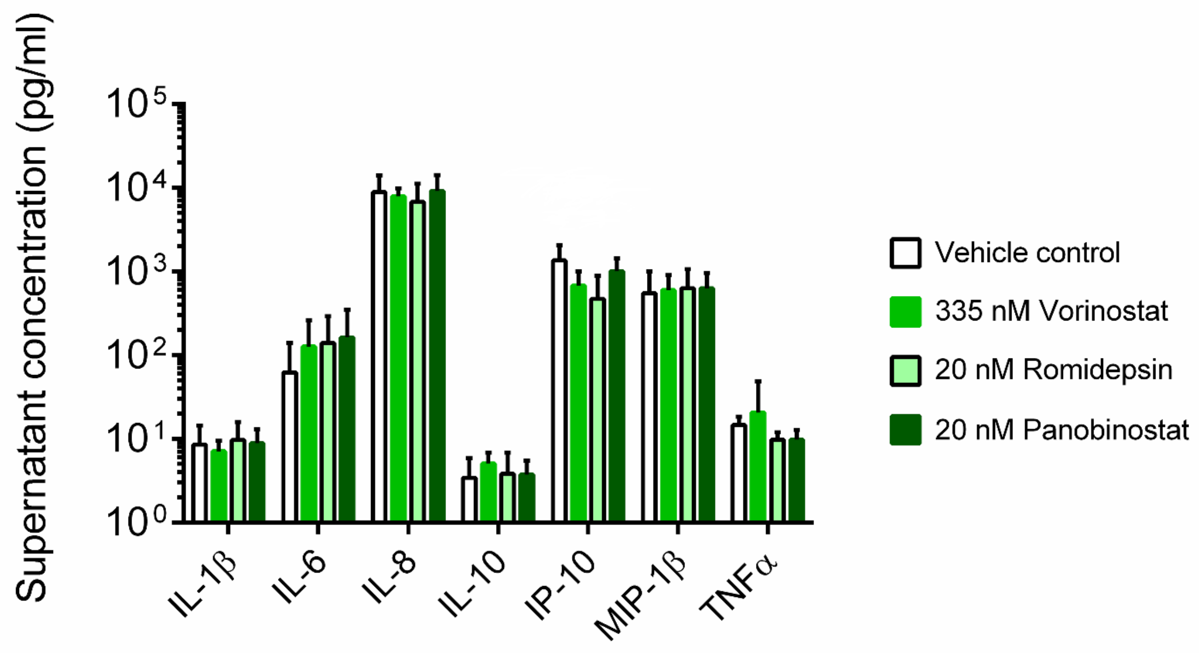

Supplementary Figure S9

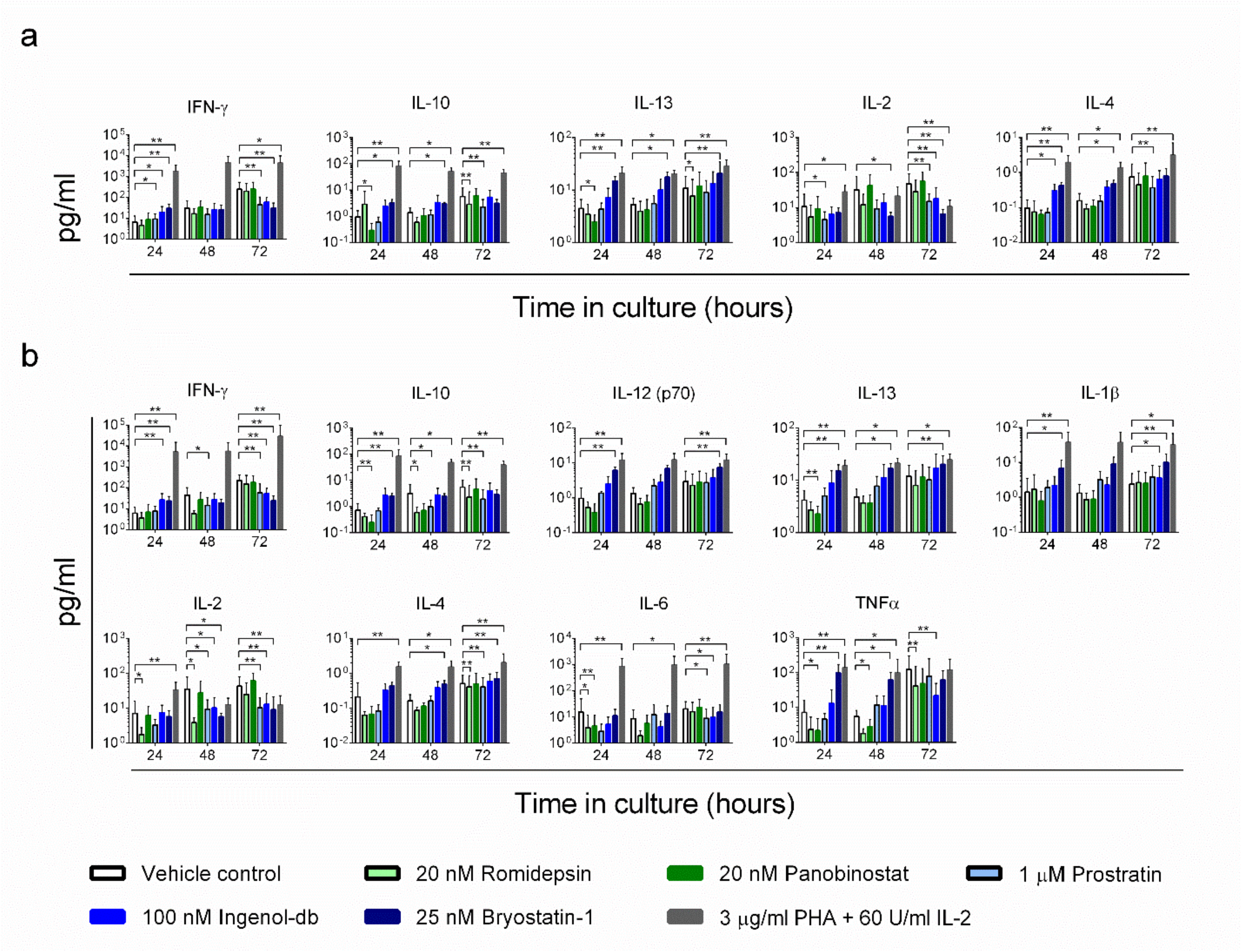

Supplementary Figure S10

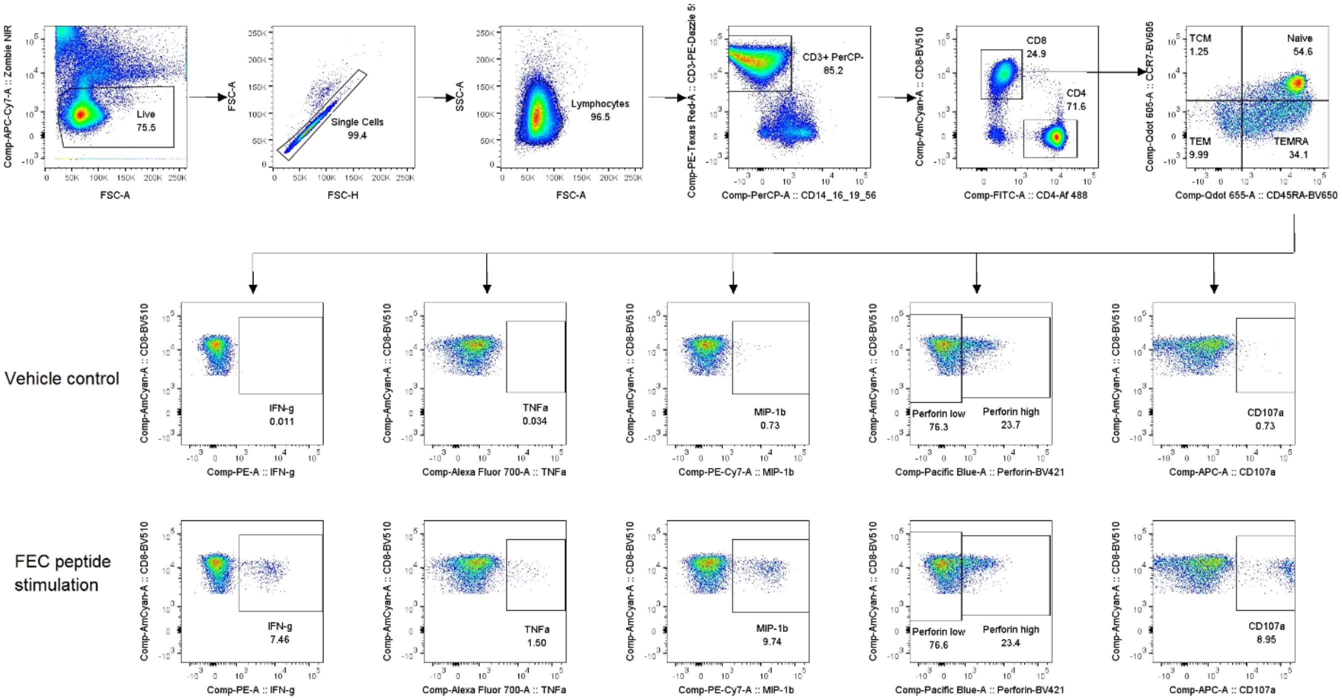

Supplementary Figure S11

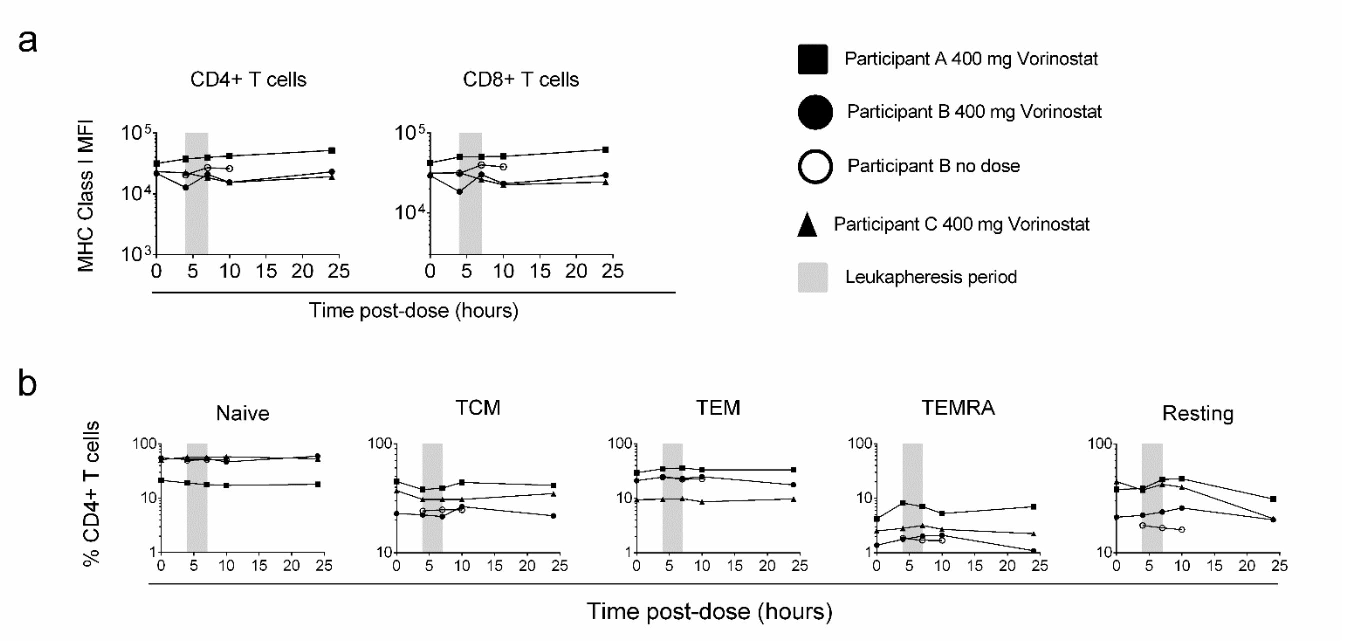

Supplementary Figure S12

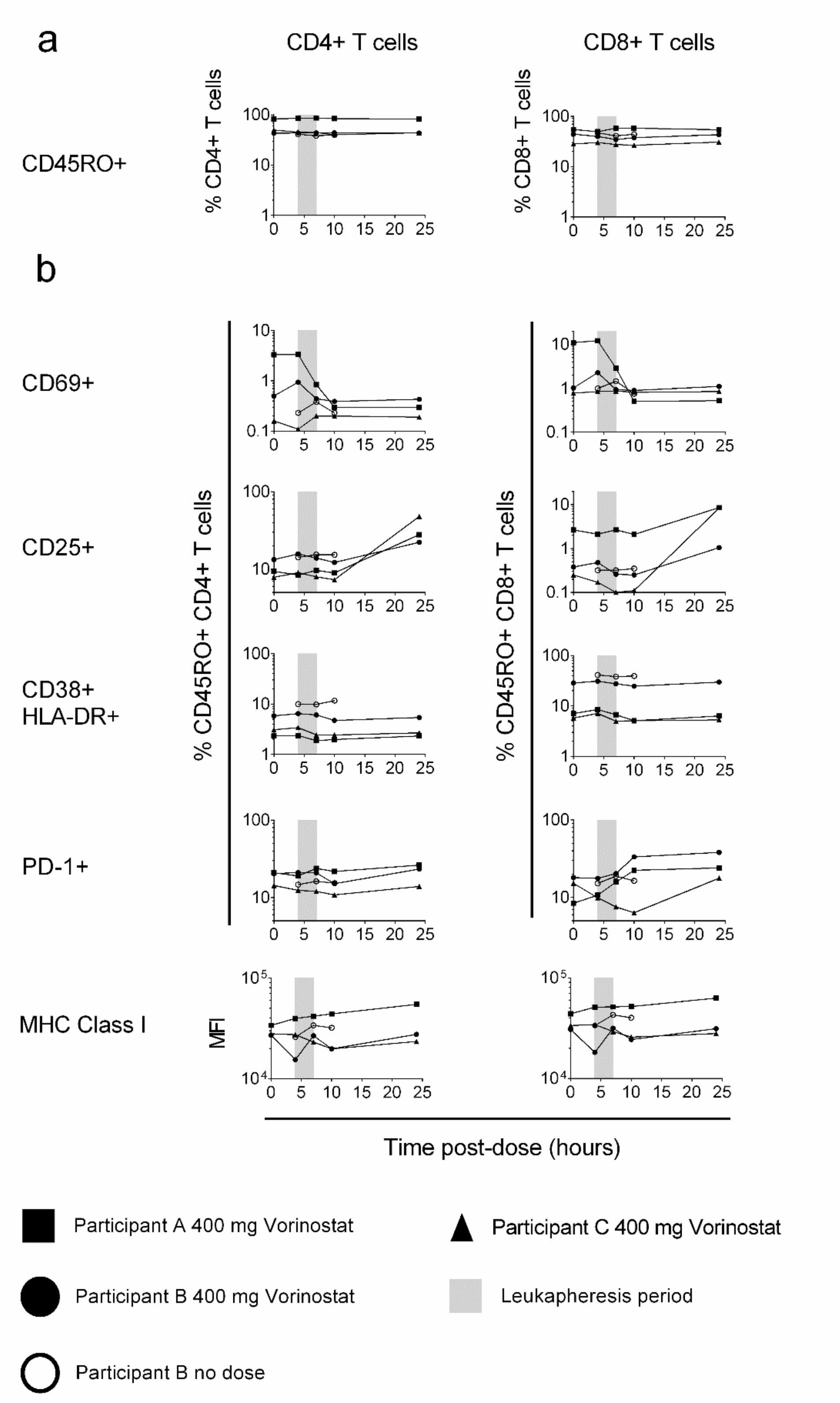

Supplement: Supplementary Information [file srep30749-s1.pdf]
